# The Virtual-Environment-Foraging Task enables rapid training and single-trial metrics of attention in head-fixed mice

**DOI:** 10.1038/s41598-018-34966-8

**Published:** 2018-11-26

**Authors:** Martha N. Havenith, Peter M. Zijderveld, Sabrina van Heukelum, Shaghayegh Abghari, Jeffrey C. Glennon, Paul Tiesinga

**Affiliations:** Donders Institute for Brain, Cognition and Behaviour, Kapittelweg, 29 6525EN Nijmegen The Netherlands

## Abstract

Attention – the flexible allocation of processing resources based on behavioural demands – is essential to survival. Mouse research offers unique tools to dissect the underlying pathways, but is hampered by the difficulty of accurately measuring attention in mice. Current attention tasks for mice face several limitations: Binary (hit/miss), temporally imprecise metrics, behavioural confounds and overtraining. Thus, despite the increasing scope of neuronal population measurements, insights are limited without equally precise behavioural measures. Here we present a virtual-environment task for head-fixed mice based on ‘foraging-like’ navigation. The task requires animals to discriminate gratings at orientation differences from 90° to 5°, and can be learned in only 3–5 sessions (<550 trials). It yields single-trial, non-binary metrics of response speed and accuracy, which generate secondary metrics of choice certainty, visual acuity, and most importantly, of sustained and cued attention – two attentional components studied extensively in humans. This allows us to examine single-trial dynamics of attention in mice, independently of confounds like rule learning. With this approach, we show that C57/BL6 mice have better visual acuity than previously measured, that they rhythmically alternate between states of high and low alertness, and that they can be prompted to adopt different performance strategies using minute changes in reward contingencies.

## Introduction

Attention – the ability to flexibly allocate processing resources based on behavioural demands – is a crucial survival mechanism, and its neuronal underpinnings in health and disease have been subject to extensive research. In human research, attention is understood as a highly differentiated process, encompassing several distinct functional sub-components, which are in turn associated with specific neuronal networks, neuromodulatory pathways, and oscillatory signatures^1–7^. For instance, the Attention Network Task (ANT) framework, developed on the basis of fMRI recordings in humans performing a range of attention-related tasks, identifies three separate components of attention^1,7–10^:

1. *Sustained Attention/Alertness*: Maintaining an alert state of sensory processing; mainly supported by thalamus, locus coeruleus, frontal and parietal cortices, with neuromodulatory signaling by the norepinephrine system^11^.
2. *Goal-driven attention/Orienting*: Orienting sensory processing towards behaviourally relevant inputs (and ignoring potential distractions); reliant on frontal eye fields and parietal lobes, and supported by cholinergic signaling^12^.
3. *Executive attention/Conflict resolution*: Reconciling conflicting inputs through top-down prioritization, and selecting responsive action through error detection and motor control; involving anterior cingulate cortex and lateral frontal cortex, with input from anterior insula and the ventral tegmental dopamine system^13–15^.

While research in humans has uncovered neuronal structures that are active during attentional processing, their causal role in generating and directing attention can only be fully tested by invasive neuronal recording and manipulation. By allowing us to directly probe the interactions between neuronal populations, tools like optogenetic actuators^16–20^, genetically expressed calcium/voltage indicators (GECIs/GEVIs)^21–26^, and designer receptors activated exclusively by designer drugs (DREADDs)^27–29^ have been a main driver of progress in this regard. As the mammalian species most suitable to these techniques, mice have become a dominant model for mapping neural circuit dynamics underlying sensory and cognitive processing^30^. Although tasks for mice compatible with neuronal recording and manipulation have drastically expanded recently (see e.g.^31–34^), tasks specifically testing attention in mice still pose a considerable challenge.

Most attention tasks currently available for mice were originally developed in the context of behavioural and clinical research rather than systems neuroscience. As such, they quantify clinically relevant aspects of attentive behaviour in a standardized way, often in an environment (e.g. an operant conditioning box) optimized for high-throughput training of animals. One classical example is the 5-choice-serial-reaction-time-task (5CSRTT), which has been a highly popular global attention test for mice – both in clinical^35,36^ and fundamental research^37,38^. In the 5CSRTT, visual stimuli appear in a random sequence across five locations, signaling the availability of reward at that location^39,40^. To succeed, animals need to constantly monitor potential target locations, making the 5CSRTT a test of *Sustained Attention* in terms of the ANT framework. The task yields standardized scores of sustained attention, impulsivity and perseverance, quantified by the number of omitted responses, premature responses, and repeated target choices, respectively. Other paradigms like continuous-performance and change-detection tasks implement variations of the same principle, requiring animals to track an ongoing sequence of stimuli, and to report (e.g. through licking or nose poke) when a target stimulus appears or when stimulus properties change^41–44^. The widespread adoption of these tasks in mice can be attributed to the way they circumvent cognitive limitations that are essentially unrelated to attention: Functions like behavioural flexibility, memory, reward and punishment learning, as well as the ability to inhibit actions, are all quite restricted in mice. As such, an ideal task for mice would test attention with minimal reliance on such other cognitive processes. Apart from simple rule memorization (‘Respond when a target appears’), sustained-attention tasks rely largely on stimulus detection, placing a low load on other cognitive resources like memory.

Other components of attention can also be tested in mice. *Goal-Driven Attention* has been tested through distractor or flanker tasks – either within the visual domain^45,46^ or across modalities^47,48^. These tasks require animals to perform an operant-conditioning task while minimizing the influence of irrelevant (distractor or flanker) stimuli. *Executive Attention* has mainly been measured by set shifting tasks, in which targets are defined along two independent stimulus dimensions (e.g. odour versus location, or shape versus brightness)^49^. The animal then has to choose a target according to one of the stimulus dimensions, signaled by a cue at the trial start^50–52^.

When applied within the context of systems neuroscience, and combined with neuronal recording, all these currently available attention tasks present several challenges. Firstly, their training schemes often prioritize high throughput and standardization over learning speed, requiring two to eight weeks of training e.g.^43,46,53^. While the issue of prolonged training has rarely been tackled explicitly, even a few hundred trials can considerably alter perceptual processing^54–56^ and fundamentally reorganize the neuronal pathways involved^57,58^. Such changes make it difficult to generalize findings from over-trained animals to other contexts. Set-shifting tasks are particularly affected: Unlike primates, who can accomplish rule shifts within minutes^59–62^, mice often require roughly as much training (~2 weeks) per shift as for a completely new task^53,63^. This suggests that at least in current vision-based paradigms, mice are essentially encoding a new rule each time rather than flexibly retrieving different rules, making their performance qualitatively different from that of other species (for a demonstration of how the VEF task tackles this problem, please see Havenith *et al.* (under review)).

Secondly, most tasks work with freely moving animals – which does not preclude neuronal recordings^64–68^, but does make recording considerably more demanding than in head-fixed animals (requiring the use of swivel-commutator systems in tethered animals or telemetric systems with large headstages and low recording capacity). It can also result in rather low numbers of trials per session – often in the tens of trials^63^. Low trial numbers in turn prohibit the use of quantification approaches most compatible with neuronal recordings.

Most importantly, since attention tasks for mice were generally not designed with the time scales of neuronal activity in mind, they largely fail to measure behavior in a precisely timed way. Most feature several seconds per trial in which the animal may or may not have made its behavioural choice - a lifetime in terms of neuronal population activity. Therefore, while these paradigms allow us to establish important links between neuronal activity and behavioural responses on average (e.g.^38,69^), they are not optimally suited to directly link specific patterns of neuronal activity to ongoing stimulus processing or decision making.

**Figure 1:**
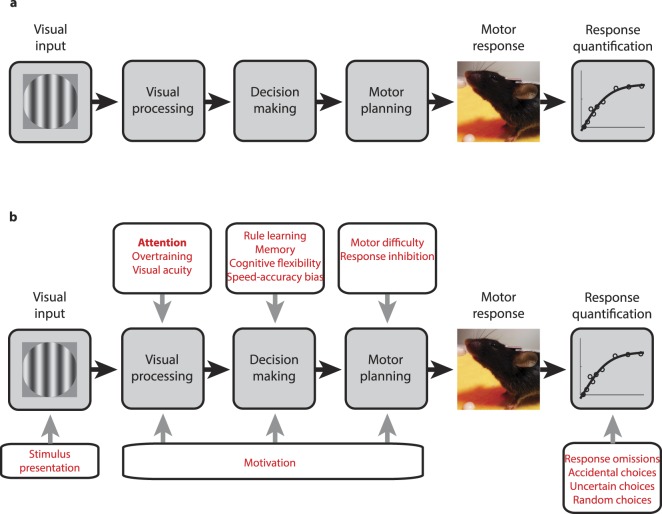
Behavioural tasks. (**a**) Schematic of the processing steps involved in completing a typical behavioural task, leading from visual input via visual processing, decision making, motor planning and motor execution to a quantified motor response. (**b**) Same as a, showing prominent factors impacting the different steps of task processing. Some of these factors are in the hands of the experimenter (e.g. stimulus presentation and the quantification of response omissions), while others are sensory and cognitive processes or capacities inherent to the animal completing the task (e.g. visual acuity and rule learning).

In addition, classical attention tasks generally fail to provide more than a binary (hit/miss) response classification. Although averaged scores across a session can yield overall estimates of e.g. impulsivity and attention, each individual trial response is only measured in terms of correct/incorrect/missing. This essentially treats attention as a discrete rather than continuous cognitive process. As a result, on a trial-by-trial basis it is difficult to exclude a vast range of confounds (see Fig. 1). Similarly, set shifting tasks struggle because binary response metrics are generally not sufficient to disentangle whether in a particular trial an animal was unable to perform an attentional shift, rather than e.g. being frustrated or confused by the task rules. Thus, while attention metrics derived from average scores are helpful and have yielded important insights into the neuronal underpinnings of attentive behaviour^38,70^, failing to quantify single-trial performance in detail squanders one of the great advantages of working with mice: Being able to concisely relate behaviour to neuronal activity moment-by-moment.

Some of these issues can be remedied by adapting tasks from more highly controlled paradigms designed for primates (e.g.^71–73^), which typically feature head fixation and exact spatiotemporal stimulus control, as well as strict time windows for trial initiation and stimulus responses. To translate such tasks to rodents, responses by saccade or button press are often converted to response by licking for reward, while the underlying task scheme largely remains the same (e.g.^74–76^). The resulting paradigms lend themselves much more easily to head fixation, which in turn ensures controlled stimulus presentation. Since they were designed for subjects that can respond to more complex cognitive demands, these tasks tend to enforce more rigorous behavioural parameters, e.g. a ‘fixation’/no-lick period at the trial start. The resulting behavioural metrics, although mostly still binary (hit/miss), are well-controlled and often more precisely timed. While such tasks have so far mostly been used to measure visual function in mice (see e.g. the psychophysical curves of contrast perception generated by Histed *et al*.^77^), they could be adapted to address higher cognitive processes by adding task components like cueing. Nevertheless, there is a price to pay for more rigorous task design: The training necessary to enforce strict behavioural limits on a mouse can require weeks to months^74,77–79^, thereby contributing further to the issue of over-training. It can also make behavioural outcomes hard to interpret: Since animals struggle to adapt to the training framework itself, it may be difficult to pinpoint whether failure to perform stems from an inability to process the stimulus or e.g. lacking motivation due to the general difficulty of inhibiting behaviours like licking.

These difficulties in satisfactorily adapting attention tasks for mice point to a more fundamental issue with behavioural paradigms (Fig. 1a): In essence, all behavioural tasks, no matter what they are designed to measure, engage multiple stages of cognitive processing from sensory stimulus processing to decision making and motor planning. These are then further augmented by experimenter-driven components of the task – how and which stimuli are presented and how motor responses are quantified. Each of these steps is shaped by multiple factors unrelated to the cognitive construct under investigation, e.g. attention (Fig. 1b). Such ongoing confounds range from general motivation, rule learning, motor abilities, to even the question if the animal was looking in the right direction when the stimulus appeared. In human paradigms, a lot of these factors can be either eliminated by explicit instruction (e.g. ‘Fixate the center of the screen’) or subsequently accounted for through self-report (e.g. rating the certainty with which a choice was made). Yet, in paradigms for animals in general and particularly for rodents, such controls are mostly unavailable. As a result, potential confounds are rarely considered or addressed explicitly (Table 1).

**Table 1:** Summary of how attention tasks for mice address behavioural confounds.

Potential confound:	5CSRTT	CPT	SST	VEF task
Stimulus presentation	/	Minimized	/	Minimized/Measured
Visual acuity	/	/	/	Measured/Eliminated
Overtraining	/	/	/	Minimized
Rule learning	/	/	/	Reduced/Measured
Memory	Minimized	Minimized	/	Minimized
Cognitive Flexibility	Minimized	Minimized	/	Minimized
Speed-accuracy bias	/	/	/	Reduced/measured
Motor difficulty	/	Minimized	/	Measured
Response inhibition	/	/	Eliminated	Eliminated/Measured
Motivation	/	/	/	Reduced/Measured
Response omissions	Measured	/	Eliminated	Eliminated
Uncertain/random/accidental choices	/	/	/	Eliminated/Measured

This table summarizes how the VEF task presented here addresses the typical behavioural confounds shown in Fig. 1, compared to three representative and popular attention tasks for mice: the 5-choice serial reaction time task (5CSRTT), the continuous performance task (CPT) and the set switching task (SST). Since there are different implementations of these tasks, we assume that the 5CSRTT and the SST are implemented in an operant conditioning box, while the CPT is implemented in a more controlled environment – with animals being head-fixed and responding with a lick/lever press response. We have categorized different confounds as either reduced, minimized, eliminated or measured (which would allow for post-hoc control). The rationale for each of these classifications is set out in Supplementary Note 1.

Virtual environment tasks^33,80,81^ have successfully combined the strengths and addressed some drawbacks of previous paradigms: By allowing for naturalistic movement in a visual space, they remove behavioural restrictions and exploit intuitive associations (e.g. ‘approach a rewarded object’), leading to shortened training and reduced learning difficulty. Behavioural responses can nevertheless be monitored precisely, animals are head-fixed, and the stimulus environment is fully controlled. As such, this approach offers interesting outlooks for faster and more versatile task training, and for enhanced trial-by-trial quantification of behaviour.

Based on a virtual-environment approach, we aimed to design a task that would tap into innate behaviour to deliver efficient training, minimize frustration and avoid ‘superstitious’ decision making in mice^82^ (e.g. alternating target choices irrespective of stimulus identity). In addition, we wanted to create a paradigm in which behaviour could be continuously monitored, generating exactly timed, nuanced performance metrics. With these metrics, we aimed to design a framework that directly addresses the confounds shown in Fig. 1, eliminating or minimizing some and explicitly quantifying others (for details, see Discussion). To meet these aims, we created a visual discrimination task for head-fixed mice, based on foraging-like navigation towards visual targets in a virtual environment. Given its reliance on innate foraging-for-reward behaviours, we refer to this paradigm as the Virtual-Environment-Foraging (VEF) task. After minimal training, the VEF task delivers two complementary metrics of attentive behavior (sustained and cued attention), which are specific, temporally precise and can be dissociated from other cognitive processes like task learning by making use of the extensive associated analysis toolbox introduced here.

## Results

**Figure 2:**
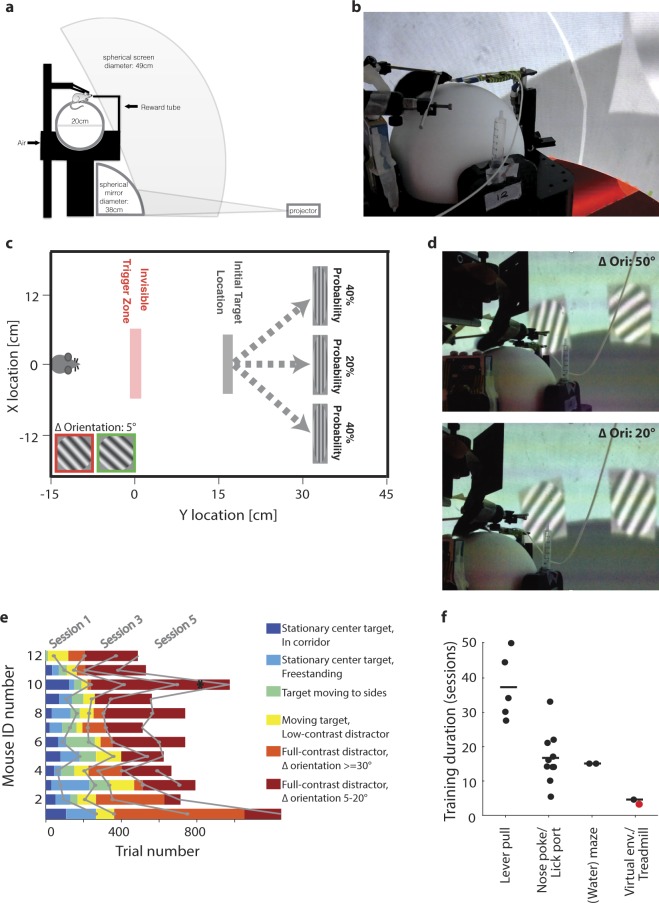
Task setup. (**a**) Schematic of experimental setup. (**b**) Image of mouse performing the task. Visible are head-plate holders, reward delivery tube, and a portion of the virtual environment projected onto the dome screen. (**c**) To-scale schematic of the virtual environment task, with virtual coordinates converted to the distance run on the treadmill. When the animal crosses the trigger zone, a zero-contrast target at the centre moves to the left, middle or right and displays a circular sinusoidal grating. When a target moves left or right, distractors displaying a differently oriented grating move in the opposite direction. Inset: Illustration of the most difficult stimulus condition, featuring a 5° orientation difference between target (framed green) and distractor grating (framed red). (**d**) Two screen shots of a mouse performing the task, with target and distractor in view. Top panel: 50° orientation difference. Bottom panel: 20° orientation difference. (**e**) Training progress of 12 animals as a function of the number of trials completed. Training is colour-coded in six stages (see inset legend). After training, animals typically completed 2–5 sessions of the task (i.e. training stage 6). Here, the first two sessions of stage 6 are shown for each animal. Grey lines: Transitions between training sessions. Black asterisk: Example session explored further in Fig. 3a–c. (**f**) Number of training sessions needed to reach consistent performance across 18 vision-based tasks for mice, categorized by response scheme (systematic literature review, see Supplementary Methods). Red dot: The VEF task. Black dots: other tasks. Black lines: Average training duration for tasks in each category.

The VEF task presented here is based on a spherical virtual environment setup, adapted from the one described by^83^. Animals were food-deprived and head-fixed on a floating-ball treadmill surrounded by a projection dome covering 270**°** of visual angle (Fig. 2a,b). Animals were initially presented with a grey target at the centre of the virtual environment. Once the animal crossed an invisible trigger threshold on its way towards the target, it would cause the target to move to the left (40% of trials), centre (20%) or right (40%), and display a circular sinusoidal grating. Centre trials simply required the animal to keep running straight ahead, and were not analysed further. When targets moved to the side, a distractor simultaneously moved to the contralateral location and displayed a competing grating of different orientation (Fig. 2c,d). In the easiest discrimination trials, targets were horizontal gratings, while distractors were vertical gratings (90**°** orientation difference). The hardest discrimination trials featured a 42.5**°** target and a 47.5**°** distractor (5**°** difference; see inset in Fig. 2c). Animals were rewarded with a cue tone and soymilk when they touched the target. If they touched the distractor instead, they would hear a punishment tone and enter a time-out corridor before restarting the same trial. Like centre-target trials, repeat trials were implemented for instruction, and not analysed further. Note that this was not a forced choice paradigm - animals could run between targets, in which case they would enter the time-out corridor without punishment tone (for details, see Methods).

The training scheme applied to entrain the VEF task was developed according to seven principles of task design for mice, described in detail by Havenith *et al*. (in this issue) Havenith *et al.* (under review). In short, we optimized learning speed and task performance by minimizing physical discomfort, reducing stress, replacing aversive punishments with trade-offs, and capitalizing on innate behaviours. Animals were trained in seven steps (see Methods; see also Havenith *et al.* (under review)). Figure 2e shows the distribution of these training steps across sessions, and the number of trials required for each training step. Animals learned to discriminate vertical and horizontal gratings within 3.4 ± 1.4 sessions (Mean ± St.Dev., corresponding to 281 ± 102 trials) from first contact with the setup, and within just 0.8 ± 0.4 sessions (87 ± 36 trials) of orientation discrimination training (training stage 4). To reach correct discrimination of gratings at orientation differences (**Δ**Ori) of ≤ 20**°**, animals required 4.7 ± 1.9 sessions (334 ± 109 trials) from first contact with the setup, and 2 ± 1.2 sessions (146 ± 81 trials) of discrimination training. This is ~20–90% faster than typical vision-based tasks for mice currently in use (Fig. 2f). It compares particularly favourably in terms of precise orientation discrimination, which is not attempted in most visual discrimination tasks for mice^33,75,79,84,85^, but when attempted tends to result in long training times and high drop-out rates^74,77,78^.

### Behavioural analysis

**Figure 3:**
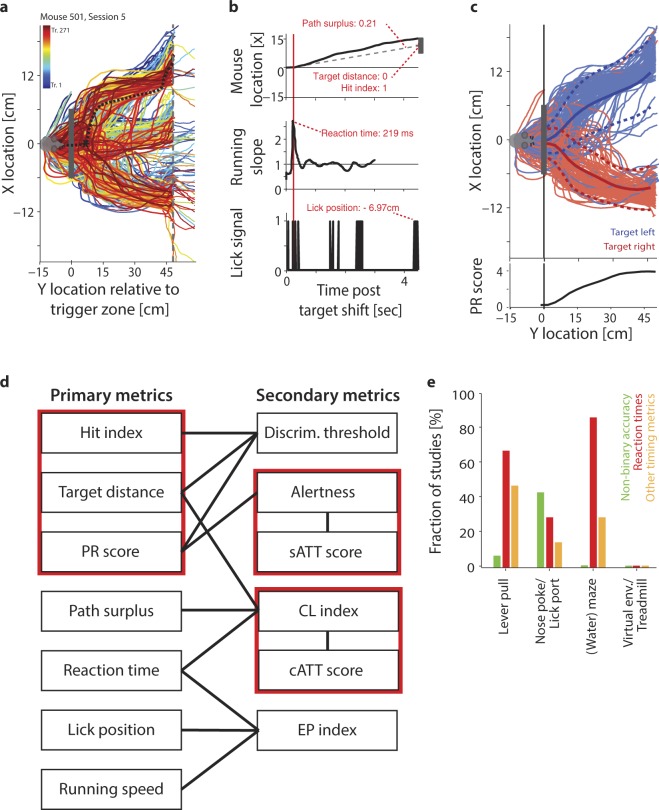
Behaviour is accurately captured by single-trial metrics. (**a**) Running trajectories through the virtual environment from the training session marked with a star in Fig. 2e, comprising 271 trials. Warmer colours represent trials later in the session. Mouse icon: Reset position at the beginning of each trial. Grey rectangle: Trigger zone for target shifts. Black dashed line: Running path analysed in panel B. Grey dashed line: Y location of targets. Trials end when the animal touches the target, the distractor, or the edge of the environment. Thus, running paths extending further than 48 cm denote trials when animals chose neither target nor distractor. (**b**) Top panel: Running path highlighted in panel a, showing lateral position as a function of time rather than Y location. Grey rectangle: Position of the target, used to compute the lateral target distance. Dashed line: ‘Ideal’ linear path if the mouse moved directly towards centre of target after decision point, used to compute the path surplus. Centre panel: Change in running slope for the same trial, showing a clear deflection shortly after the target displacement. Red line: Time of largest deflection in running slope, used to measure the reaction time (219 ms). Bottom panel: Trace of licking for the same trial, with each lick denoted as 1. (**c**) Top panel: Same as A, but showing only trials for left and right target positions. Blue: Running trajectories for trials with left-hand targets. Red: Trajectories for right-hand targets. Dark lines: Mean path per target location. Dark dashed lines: Mean path ± 1 Std.Dev. Bottom panel: PR score measuring the reliability of running trajectories per target location, computed for each Y location as the normalized difference between the X locations for left and right targets (see Methods). (**d**) Schematic of primary and secondary performance metrics. Primary metrics are derived as shown in panels b-c. Secondary metrics are derived from primary metrics as shown by connecting black lines. Red boxes surround metrics that are mathematically interdependent. (**e**) Fraction of recent vision-based paradigms for mice that utilized performance metrics beyond a basic hit/miss trial classification. Each metric employed by a study was categorized either as a non-binary metric of accuracy, as reaction times or as other timing measures (e.g. time-to-target). Only a minority of tasks employs any advanced performance metrics, and the tasks that yield non-binary accuracy metrics virtually never yield response timing metrics (and vice versa). The shown fractions are based on 31 vision-based tasks for mice published between 2011 and 2016 (see Supplementary Methods) rather than attention tasks specifically, simply because attention tasks for mice are even rarer and use virtually exclusively binary hit/miss classifications.

Because animals are head-fixed in a virtual environment, the VEF task generates a continuous stream of well-controlled readouts of running and licking. While we did not record eye and whisker movements in the current configuration, such readouts can easily be added if necessary. Such continuous behavioural tracking allowed us to create multiple nuanced (non-binary) single-trial measures of the timing, accuracy and reliability of task responses. Figure 3a–c illustrate how behavioural measures were extracted from each trial. In total, we extracted seven primary metrics of response accuracy and speed per trial (Fig. 3d): (1) As a simple metric of accuracy, we computed a hit index, encoded as 1 for correct trials, 0 for undecided trials, and -1 for trials when the animal approached the distractor. (2) As a non-binary metric of accuracy, we measured the animal’s lateral distance from the target at trial offset. The target distance was normalized by the distance between target positons to make it independent of the specific dimensions of the virtual environment. As such, a target distance of 0 represents hit trials, values close to 1 indicate cases when animals were approximately one target location removed from the target at the end of the trial (e.g. close to the centre target position when the actual target was located on the right), and values close to 2 indicated animals ending up two locations away from the target (e.g. running to the left when the target was on the right). (3) To represent the reliability of responses beyond hit/miss classifications, we computed the path reliability (PR) score, which quantifies the reproducibility of running paths towards the same target using the effect size Cohen’s D (Fig. 3c). The PR score decreases in the presence of error trials, and increases with hit trials, but also with the reproducibility of successful running trajectories. Note that hit index, target distance and PR score are to some extent correlated by mathematical necessity, since all three are jointly affected when an animal succeeds or fails to reach the target. (4) To quantify not only the accuracy of the final target choice, but also the efficiency with which animals moved towards their chosen target, we computed the ‘path surplus’, defined as the length of the actual path taken, normalized by the ‘ideal’ path - if the animal had directly approached the chosen target (i.e. the one it ended up closest to) after the point of decision making (Fig. 3b, upper panel). This metric increases in trials in which animals ‘change their mind’ – moving first towards one target, then changing direction towards the other. (5) As a metric of reaction time, for each trial we determined the most abrupt change in running direction and used it to define the moment of target choice (Fig. 3b, centre panel). (6) As a proxy for reward anticipation, we computed the average Y position at which animals licked for reward within the vicinity of the target (Fig. 3b, lower panel; see also Havenith *et al.* (under review) for more detailed analyses of anticipatory licking). (7) Finally, as an indicator of motivation and a warning signal of potential motor impairments, we recorded the average running speed following the target shift. Details on all primary metrics can be found in Methods (for four auxiliary metrics, see Supplementary Methods). Supplementary Figure S1 provides an example of the metrics described above, extracted from the recording session shown in Fig. 3a–c.

**Figure 4:**
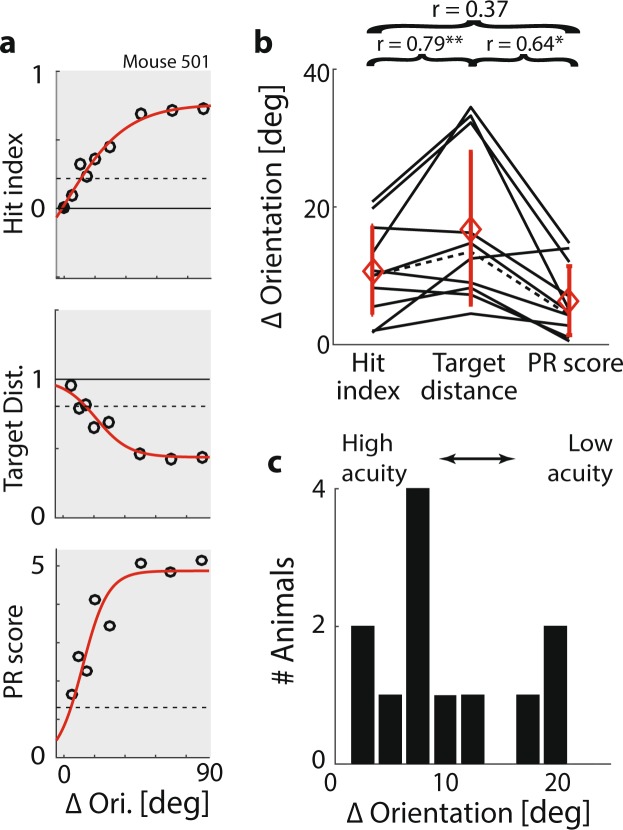
Measuring visual acuity. (**a**) Top panel: Average hit index as a function of ΔOri for one animal. Circles: Average performance per ΔOri. Red line: Fitted sigmoid function. Black line: Chance level. Black dashed line: Criterion value for significant orientation discrimination based on a bootstrapped estimate of error variance (α = 0.05, see Methods). Centre and bottom panel: Same for target distance and PR score, respectively. (**b**) Visual thresholds for 12 animals, estimated based on the psychometric curves of hit index, target distance and PR score, as shown in A. Black lines: Animals. Dashed line: Animal shown in A. Red lines: Mean ± Std.Dev. Correlations between the visual thresholds derived from hit index, target distance and PR score are shown above the panel (*p < 0.05; **p < 0.01). (**c**) Distribution of overall visual thresholds across 12 animals. Each visual threshold was computed by averaging the thresholds derived from hit index, target distance and PR score (shown in panel b).

**Figure 5:**
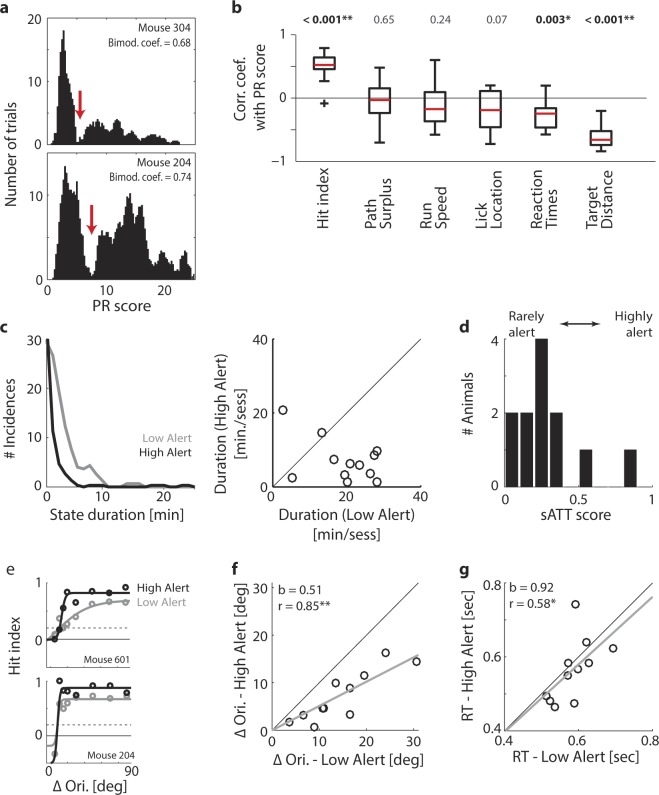
Sustained attention fluctuates rhythmically. (**a**) Distribution of local PR scores for two mice, across all sessions for the final training stage. Local PR scores were computed for sliding windows of 15 trials. Red arrows: Transition point between the two modes of the distribution, determined by Larkin’s F-test (see Methods). Note that although the distributions differ in shape, they can both be classified as bimodal according to their Bimodality Coefficient (Bimodality threshold: Bimod. Coeff. = 0.55; see Methods). (**b**) Box plot of correlation coefficients between local PR scores and the running averages of the other six primary metrics, computed per animal. Red line: Median. Rectangle: 1st and 3rd quartile. Whiskers: 1st and 9th decile. Crosses: Outliers. (**c**) Left panel: Duration of individual High- and Low-Alert states. Grey: Low-Alert episodes (n = 161). Black: High-Alert episodes (n = 137). All sessions from training stage 5 onwards are included (n = 65 sessions in 12 animals). Right panel: Average time per session spent in High- and Low-Alert states. Dots: 12 animals. Line: Unity line. (**d**) Distribution of the sATT score across 12 animals, computed as the ratio between the time per session occupied by high- and low-alert states. (**e**) Psychophysical curves for two animals, computed for trials recorded during High-Alert (black) and Low-Alert states (grey). Circles: Average hit index per ΔOri. Lines: Fitted sigmoid function used to determine the visual discrimination threshold. Dashed line: Critical value for discrimination threshold (α = 0.05; see Fig. 4a and Methods). (**f**) Scatter plot of the visual thresholds derived from trials in Low- and High-Alert phases. Circles: 12 animals. Black line: Unity line. Grey line: Fitted linear regression, with the linear offset fixed to zero. r: Correlation coefficient. b: Slope of the regression. (**g**) Same as panel f for reaction times.

**Figure 6:**
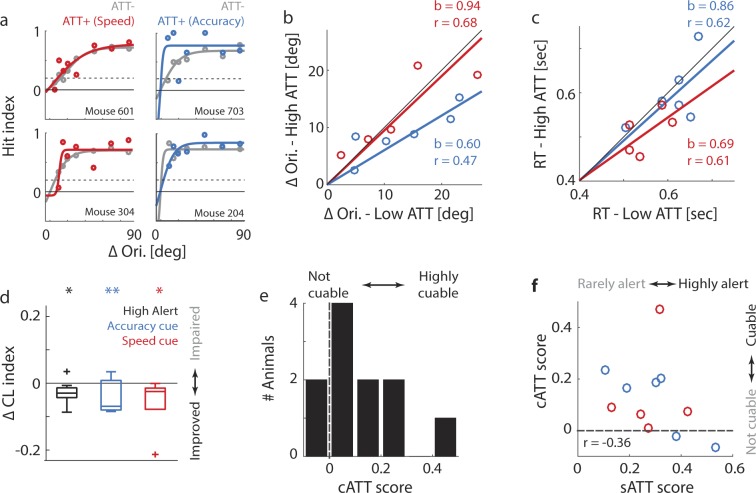
Cued attention: Animals can be cued to increase response speed or accuracy. (**a**) Same psychophysical curves as Fig. 5e, for CUE and Non-CUE rather than High- and Low-Alert trials. Grey: Non-CUE trials. Blue: CUE trials with accuracy incentive (see Methods). Red: CUE trials with speed incentive. (**b**) Same as Fig. 5f for CUE and Non-CUE rather than High- and Low-Alert trials. Blue: Accuracy incentive. Red: Speed incentive. (**c**) Same as Fig. 5g for CUE and Non-CUE rather than High- and Low-Alert trials. Blue: Accuracy incentive. Red: Speed incentive. (**d**) Distribution of changes in the Cognitive Load (CL) Index between attentional states. Rectangle: 1st and 3rd quartile. Centre line: Median. Whiskers: 1st and 9th decile. Crosses: Outliers. Black: High-Alert versus Low-Alert trials (n = 57 sessions in 12 animals). Blue: CUE versus Non-CUE trials (accuracy incentive; n = 30 sessions from 6 animals). Red: CUE versus Non-CUE trials (speed incentive; n = 27 sessions from 5 animals). Asterisks: Statistical significance of performance changes based on a Repeated-Measures ANOVA (*p < 0.05; **p < 0.01). (**e**) Distribution of the cATT score across 11 animals that received cued trials, computed as the difference between average CL indices for CUE versus Non-CUE trials, normalized by the average CL index across all trials. (**f**) Scatter plot of sATT scores versus cATT scores for 11 animals. Blue: Animals receiving the accuracy incentive (n = 6). Red: Animals receiving the speed incentive (n = 5).

Based on these primary metrics, we computed secondary behavioural metrics geared towards quantifying specific cognitive processes. This is a similar process as converting action potentials, which are derived quite directly from raw recordings of neuronal activity (primary metrics) into more global measures of neuronal activity, for instance firing rates, synchronization or phase locking (secondary metrics). We derived six secondary metrics, which will be examined in detail in Figs 4–6. Briefly, the six metrics were constructed as follows: (1) As a measure of visual accuracy, we computed each animal’s visual threshold of orientation discrimination based on the psychometric curves of hit index, target distance and PR score (see Fig. 4). (2) We derived a measure classifying spontaneous ‘up and down states’ of alertness based on the bimodal distribution of local (15-trial sliding average) PR scores over time (see Fig. 5). (3) Based on the classification of attentional up and down states, we computed a metric of sustained attention referred to as the sATT score, which represents the proportion of time an animal succeeded in maintaining a state of high alertness (see Fig. 5). (4) Consistent with a large body of literature on the trade-off between performance speed and accuracy across species from humans to insects^86–90^, different mice seemed to show different behavioural priorities, preferring to adjust either their speed or accuracy in difficult trials (see Fig. 6). To compare animals’ overall performance irrespective of performance style, we generated the Cognitive Load (CL) index. The CL index was computed as the normalized sum of response speed (reaction time), response efficiency (path surplus) and response accuracy (target distance), increasing when animals were responding slowly, inefficiently or incorrectly. (5) Based on the CL index, we created a second metric of attention referred to as the cATT score. In contrast to the sATT score, the cATT score is geared towards measuring ‘orienting’, goal-directed attention. It does so by quantifying to what extent an animal’s CL index improved in cued trials, which offered increased reward and punishment based on task performance. The difference of CL index between cued and non-cued trials was then normalized by the average CL index across all trials, such that for instance a cATT score of 0.5 would signal a 50% improvement of the CL index in cued trials compared to non-cued ones. (6) By comparing reaction speed, running speed and anticipatory licking in hit versus miss trials, we computed an index of error prediction that reflected whether animals showed reduced response certainty and reward anticipation in incorrect trials. Such a reduction would indicate that an animal had in fact internalized the task rule and was therefore able to predict whether or not a response was correct and therefore likely to result in reward. Most importantly, this metric allowed us to quantify rule comprehension independently of rule execution. The implications of this division, and further analyses of rule acquisition, are presented in detail elsewhere Havenith *et al.* (under review).

Graded, single-trial metrics of behaviour like the ones introduced above have so far been difficult to achieve in other paradigms - even in other virtual-environment tasks (Fig. 3e). What’s more, changing running direction on a treadmill was sufficiently energy-consuming that animals never responded randomly in the absence of target stimuli. We tested this in two animals by removing the visual projection of the virtual environment for ~15 minutes. In the absence of a visible target, both mice never changed running direction (Supplementary Fig. S1). In other words, responses in this paradigm were free of false positives or random task responses (see Table 1) - the moment at which an animal changed running direction could be taken as a reliable marker of a deliberate target choice. This stands in marked contrast to simpler response paradigms (e.g. based on licking), in which a response is metabolically and cognitively cheap. As a result, animals often have to be specifically trained to refrain from random licking^33,74,76,78^, and at least some proportion of trials in these tasks is likely to feature unidentifiable false positives (Table 1).

Note that all performance metrics are based on directed treadmill running. This raises the concern that performance might be driven by motor processing just as much as perceptual decision making. To estimate the contribution of motor difficulty, we compared task performance for easy and difficult visual stimuli (Supplementary Fig. S2). Performance for easy stimuli was strongly clustered towards its optimum, suggesting that response variability largely originates from sensory rather than motor processing. The level to which motor processing affects task performance is thus likely comparable to more established response schemes like licking or lever press paradigms, and arguably superior to paradigms involving free running (e.g. touchscreen tasks). For a more detailed comparison of how motor processing affects different task response schemes, please see the Discussion.

### Measuring visual acuity

The primary metrics of performance accuracy introduced above - hit index, target distance and PR score – can also be used to generate psychometric curves of orientation discrimination. While obtaining a rigorous measure of visual acuity in mice is in itself a goal, and applicable to a multitude of studies of visual processing, it is also important in the context of measuring (visual) attention: In the absence of an explicit measure of visual acuity, low performance in a visual attention task cannot be unequivocally attributed to low attention (see Fig. 1b). For instance, animals with low vision would most likely show a higher number of omitted trials in the 5CRTT, leading to a lower attention score. In the VEF task, visual acuity is quantified directly, and the mutual independence of attention metrics and visual acuity is confirmed explicitly (see Fig. 5; Supplementary Fig. S3).

To quantify visual acuity, we estimated a threshold of orientation discrimination for each animal using hit index, target distance and PR score. We first defined critical values at which performance could be assumed to differ significantly from chance based on a bootstrap analysis of performance variability across all animals (see Methods). For the hit index, the criterion was 0.20; for target distance, it was 0.83; and for the PR score it was 1.25. For each animal, we then fitted the corresponding psychometric curves with a sigmoid function, and determined the ΔOri at which the curve reached the critical value (Fig. 4a). As expected, thresholds computed from hit index, target distance and PR score correlated strongly (Fig. 4b). Hit index and PR score had the lowest agreement, while target distance correlated highly with both, but tended to overestimate the discrimination thresholds for low-performing animals (ΔOri > 15°; Fig. 4b). To minimize error variance, we computed each discrimination threshold by averaging the estimates from target distance, PR score and hit index. Note that 7 of 11 animals (64%) reached discrimination thresholds below 10° (Fig. 4c). In other words, most animals achieved largely correct orientation discrimination for ΔOri ≥ 10°, and often even for ΔOri ≥ 5°. This presents a marked improvement compared to estimates of orientation discrimination in mice from more restrictive paradigms^74,78^, indicating that despite generally coarser vision^82,84,91^, in an adaptive, naturalistic task mice can identify even minute orientation differences.

### Sustained attention: Quantifying fluctuations in alertness

Sustained attention, or vigilance, is the ability to maintain alertness to relevant stimuli over extended periods of time^92^. In the VEF task, animals seemed to exhibit spontaneous rhythmic fluctuations of performance, which were most noticeable in the PR score, but also faintly visible in other metrics (see Supplementary Fig. S1). We hypothesized that such fluctuations reflected alternating states of high and low alertness, and could therefore be used to quantify an animal’s capacity for sustained attention. To test this hypothesis, we first verified that across animals, the local PR scores indeed switched between high and low phases, resulting in a bimodal distribution. Figure 5a shows local PR scores for two animals, together with the resulting bimodality coefficient. The bimodality coefficient indicated bimodal distributions for 11 of 12 animals (see Methods and^93^), confirming that local PR scores switched between high and low episodes. Given the low number of error trials in well-trained animals, apparent fluctuations of local PR scores could in principle be produced by individual error trials in otherwise error-free trial sets. We excluded this possibility by confirming that bimodality was maintained in bootstrapped data where error trials had been reassigned as hit trials (Supplementary Fig. S3).

Next, we related PR score fluctuations to the other primary performance metrics by smoothing all measures with a 25-trial sliding averaging window, and correlating the resulting traces over time (Fig. 5b). The correlation coefficients shown in Fig. 5b confirmed that performance improved across the board in trials associated with high local PR scores: Responses were faster, more accurate, and reward was anticipated earlier (t-test for difference of correlation coefficients from 0 with Dunn-Sidak correction for multiple comparisons across six metrics; df = 11 based on coefficients from 12 animals; t statistics from left to right: 4.3, -1.2; -0.1; -2.1, -2.9, -8.8; p values above Fig. 5b; bold font indicates significant differences after Dunn-Sidak correction; see Supplementary Table S1). Some cross-metric relations would be expected out of mathematical necessity. For example, the PR score will rise if there are fewer incorrect trials, and as such it is by definition related to the hit index. However, other measures that varied with High- and Low-Alert states (e.g. reaction time and lick location) were mathematically entirely independent (Supplementary Fig. S3). This suggests that most cross-measure correlations emerged not by mathematical necessity, but because underlying transitions between high and low alertness jointly impacted all aspects of performance. As such, we were able to use local PR scores to define individual trials as belonging to either High-Alert or Low-Alert phases, using a cut-off criterion derived from each animal’s unique bimodal distribution of PR scores (see Fig. 5a and Supplementary Fig. S4).

We next quantified how much time animals spent in High-Alert and Low-Alert states. High-Alert phases were generally more limited: Individual High-Alert episodes rarely lasted more than ten minutes (Fig. 5c, left panel), and all High-Alert episodes in a session together never occupied more than 25 minutes (Fig. 5c, right panel). The average portion of a session spent in High Alert was 31% ± 25% (Mean ± St.Dev.). Interestingly, this portion varied considerably across animals: While some (‘highly alert’) animals reached up to 80%, others (‘rarely alert’) only spent <20% of their time in High-Alert states (Fig. 5d; see also Supplementary Fig. S4). We refer to the proportion of time spent in High-Alert states as the sATT score. The sATT score offers a direct estimate of an animal’s capacity for sustained attention: It separates highly focused animals from less alert ones without requiring them to train any specific attention test. Instead, it simply arises from the observation of spontaneous performance fluctuations in a reasonably demanding discrimination task – in our case, a visual orientation discrimination task. However, any task that can be framed in terms of virtual-environment navigation could serve the same purpose. Moreover, the sATT score relies purely on the time spent in high alertness, not on absolute task performance. This is important because in different animals, high alertness may result in higher or lower overall performance for reasons unrelated to attention, e.g. visual acuity (see Figs 1b, 4c). By defining the criteria for high and low alertness for each animal relative to their own individual performance distribution, such confounding factors are excluded (Table 1).

Quantifying High-Alert states also proved helpful in determining the true (i.e. optimal) visual acuity of an animal: Fig. 5e shows psychometric curves based on High-Alert and Low-Alert trials for two example animals. In one case, High-Alert episodes led to sharpened visual discrimination (Fig. 5e, top panel), demonstrating that the animal was visually capable of finer orientation discrimination than its performance during Low-Alert episodes would have suggested. In the other example, the discrimination threshold only changed marginally (Fig. 5e, bottom panel), indicating that the animal utilized its full visual capacity irrespective of alertness. Note that this does not imply a failure to improve - in Fig. 5e, overall performance increased in both animals during High-Alert episodes, but only in one did this shift the discrimination threshold. On average, visual thresholds decreased by 6.8° ± 4.7° (i.e. 48% ± 25%) for High- compared to Low-Alert episodes (Mean ± St.Dev.; t-test for dependent samples: df = 10 based on 11 animals that showed a binary distribution of local PR scores; t = 2.2; p = 0.05; see Supplementary Table S1). However, the magnitude of the effect varied considerably across animals (Fig. 5f), with different animals improving by 1.0° to 16.1° (or 5% to 92%). This suggests that while for some animals, increased alertness could improve visual discrimination, others presumably reached a ‘hard’ physiological limit, whereby heightened alertness could not override the maximum visual acuity they were capable of. Such an analysis can be of service to distinguish average visual discrimination performance (Fig. 4c) from an animal’s true upper limit of visual acuity (Fig. 5f). While animals also seemed to show a small (non-significant) tendency to respond more quickly during High-Alert states (Fig. 5g; t-test for dependent samples: df = 10; t = 0.52; p = 0.57; see Supplementary Table S1), performance mainly improved in terms of accuracy. This suggests that High-Alert states favoured ‘thorough’ responses, prioritizing accuracy over speed.

Together, these results indicate that local PR scores reflect genuine spontaneous fluctuations in processing capacity, resulting in cohesive changes across multiple aspects of performance. Quantifying these spontaneous fluctuations provides us with the sATT score, a concise metric of sustained attention in rodents that does not depend on long-winded training of a specific attention task, is not contaminated by confounding factors of performance like visual acuity or motor prowess, and is readily comparable to measures of sustained attention in humans^1,10,11^. Depending on the experimental design in question, this metric can be used to either identify and filter out attention-dependent fluctuations in performance (e.g. to capture optimal visual acuity), or to harness them for subsequent analyses. Such analyses could for instance aim to predict episodes of increased alertness from neural responses, or to extract neuronal network features that mark animals with a high or low capacity for sustained attention (both in wildtype populations and in mouse models of attention-related disorders).

### Cued attention: Prompting animals to prioritize response speed or accuracy

Compared to spontaneous fluctuations in alertness, the active (‘top-down’) allocation of processing resources to behaviourally relevant stimuli is thought to rely on separate attentional processes^2–6,10,12,14,60,94^. Within the ANT framework of human attention^1,8–10^, this would translate to the differentiation between Component 1 (*Sustained attention*, the ability to maintain a state of alertness^11^), and Component 2 (*Orienting Attention*, the ability to orient towards relevant sensory information^12^). To explore whether the VEF paradigm can also be used to test goal-oriented top-down attention in mice, we tackled two questions: a) Can increases in performance be actively triggered by cuing? and b) Can animals be cued to adjust their processing strategy in order to prioritize either fast or correct responses? To this end, we introduced two cuing paradigms in which animals could gain additional reward based on response accuracy or speed. In 20% of trials, an auditory cue at the beginning of the trial would signal increased reward (more soy milk) for correct responses and increased punishment (a longer time-out corridor) for incorrect responses. In the accuracy condition, reward was simply doubled in hit trials. In the speed cuing condition, the additional reward scaled with the speed with which animals reached the target, delivering anywhere from 1.5 to 4 times as much soy milk as in baseline trials. In both cases, miss trials incurred a time-out corridor of double length.

Figure 6a shows the psychometric curves of four animals cued either with the speed or accuracy incentive. Like spontaneous High-Alert states (Fig. 5e,f), cued trials also generally improved discrimination thresholds. However, while accuracy cuing resulted in similar improvements as High-Alert episodes (Fig. 6b, compared to Fig. 5f; df = 5; t = 1.54; p = 0.18), speed cuing did not greatly affect discrimination (Fig. 6a,b; t-test for dependent samples: df = 4; t = 0.94; p = 0.39; see Supplementary Table S1). In contrast, speed cuing shortened reaction times more strongly than either accuracy cuing or High-Alert states (Fig. 6c, compared to Fig. 5g; Accuracy cue: df = 5; t = 0.22; p = 0.84; Speed cue: df = 4; t = 2.42; p = 0.07; see Supplementary Table S1). This pattern was borne out across other primary performance metrics: The accuracy cue mainly improved hit index, target distance and path surplus, while the speed cue led to faster reaction times, faster running and earlier licking (Supplementary Fig. S5). In comparison, spontaneous High-Alert states seemed to act more broadly, improving both response accuracy and speed (Supplementary Fig. S5). The marked divergence of performance styles between speed-cued and accuracy-cued trials indicates that mice are able to compute intricate reward contingencies, and adjust their actions accordingly. As a result, even minute changes in a task’s reward scheme (in our case, a maximum increase of 30 μl of soy milk per trial) are enough to prompt animals to adopt different behavioural strategies (for an in-depth analysis of the dynamics of rule learning in this task, see^95^).

Interestingly, when these performance changes are viewed through the CL index – a composite measure incorporating primary metrics of accuracy and speed – spontaneous High-Alert, accuracy- and speed-cued trials all induce a similar increase in overall performance capacity (Fig. 6d and Supplementary Fig. S5). This is also supported by data shown in Supplementary Fig. S6: While animals generally demonstrated a speed-accuracy trade-off of performance comparable to those reported in previous studies^86–90^, the trade-off was reduced both in High-Alert and CUE trials. This suggests that in these contexts, overall cognitive resources were enhanced, so that they could be allocated simultaneously to accurate and speedy performance. These results showcase the utility of advanced performance metrics like the CL index in revealing underlying principles of cognitive processing: Here, the CL index allowed us to highlight the fact that task processing improved to similar extents during High-Alert and CUE episodes, irrespective of performance style.

To summarize an animal’s capacity to allocate cued attention, we defined the cATT score as the normalized difference in CL index between cued and non-cued trials (Fig. 6e). Similarly to the distribution of sATT scores (Fig. 5d), the cATT score displayed considerable inter-individual differences (from −0.1, indicating a small disruptive effect of cuing, to 0.5, indicating a 50% increase of performance capacity compared to non-cued trials), suggesting that some animals responded more strongly to cuing than others. Interestingly, an animal’s receptiveness to cuing was not predicted by its improvement during High-Alert states: When directly comparing sATT scores and cATT scores for the same animals, capacity for sustained and cued attention were hardly related at all (Fig. 6f; n = 11 animals; r = −0.36; p = 0.28). This was also confirmed when comparing performance improvements in High-Alert and CUE states directly – improvement in one context did not predict improvement in the other (Supplementary Fig. S5). These results are a strong indication that with the two metrics of sATT score and cATT score, we are indeed measuring two complementary and largely independent aspects of attentive processing, akin to the first two components of the ANT framework of attention in humans.

## Discussion

Here we present a vision-based attention task for head-fixed mice, based on foraging-like navigation in a virtual environment. By optimizing the training process, and by tracking a range of performance metrics trial-by-trial, our task meets several goals not achieved by previous paradigms. First, it yields a concise quantification for two distinct aspects of attention that have been studied extensively in humans: Sustained and cued or goal-directed attention. By tapping into innate foraging behaviour, it manages to deliver these metrics within drastically reduced training times, and without the need for specific ‘attention task’ training. This not only simplifies experimental designs, it also disentangles the assessment of attention from that of learning ability. By providing head fixation, the task is easily combined with various techniques of neuronal population recording and manipulation, including electrophysiology, two-photon imaging and optogenetics; and precisely timed behavioural tracking allows us to directly relate recorded neuronal activity to critical moments of decision making on a trial-by-trial basis. Finally, an array of single-trial performance metrics allows the VEF task to eliminate behavioural confounds that commonly go unaddressed not only by attention tasks but by most sensory and cognitive tasks for rodents (Fig. 1 and Table 1). Together, these features open up the opportunity to study the distinct neuronal networks underlying attentive processing, drawing direct trial-by-trial links between neuronal dynamics and two different components of attention.

While we focus on a non-forced two-choice task structure here, the framework is easily adapted to different experimental questions by varying the type, number, timing, spatial distribution and difficulty of visual stimuli, adding cues and varying reward rules. For example, in the present task configuration animals tended to prioritize accuracy over speed, leading e.g. to improved accuracy but largely constant reaction times during High-Alert states (see Fig. 5f,g). Such a preference was welcome here, but it could easily be addressed by modifying the task to penalize slow trials more heavily. Similarly, a forced choice could be introduced simply by adding separating walls between targets.

With this approach, we show for the first time that mice consistently alternate between states of high and low alertness over time, and that there are considerable inter-individual differences in the degree to which animals sustain high alertness. We manage to quantify such attentional capacity independently of sensory (in this case visual) acuity – a distinction that to our knowledge has not been achieved by previous attention tasks for rodents. By separating out the contributions of attention and visual acuity to task performance, we demonstrate that some animals operate at their optimal visual acuity regardless of attentional state, while other animals regularly underperform the limits of their visual system – an important insight not only for studies of attention but also for experiments aiming to quantify visual processing in mice. Moreover, we show that even small changes in reward contingencies can effectively inform performance strategy in mice. Finally, we confirm that sustained and goal-oriented attention indeed form two largely independent aspects of attentive behaviour, as evidenced by the fact that an animal’s capacity for one did not predict its capacity for the other.

One of the main advantages of this paradigm is that it reduces training times by 40–90% compared to other visual paradigms for mice. We also experience virtually no drop-out of animals during training, compared to up to 80% drop-out for tasks of similar difficulty (as far as one can determine from the rare papers that explicitly mention drop-out rates^74,76^). This opens doors to new experimental designs involving behaving animals, and removes a crucial confound: The neuronal changes triggered by long-term overtraining of a task (see e.g.^57,58^), and the fact that if up to 80% of animals cannot complete training, the remaining 20% probably have a very specific cognitive (and therefore neuronal) make-up unrepresentative of naturalistic cognitive processing in mice. Rapid training also enables the study of juvenile animals, opening up new developmental perspectives on attention in mice.

Another important advantage of our task lies in the fact that it offers a precise and versatile quantification of behaviour per trial. This allows behavioural output to be related to ongoing physiological and neuronal processes not only with better precision, but in a qualitatively different way. This principle is handily illustrated by the example of reaction times. While many rodent tasks offer measures of response timing, in practice these estimates are often not directly linked to the actual moment of decision making. For example, in most free-moving paradigms (including the 5CSRTT), the main timing measure is the time-to-target. This is not only contaminated by variables like running/swimming speed, it also hardly reflects the time at which the target choice is made. In contrast, tasks based on licking/nose poke/lever press responses can in principle give a reaction time that relates more directly to e.g. the moment of stimulus detection. However, the fact that the required response is binary, and of low cost to the animal, means that e.g. one random lick can confound the estimated reaction time for a whole trial. Moreover, since a lot of paradigms give reward at a specific delay after the stimulus presentation, animals begin to anticipate this delay, and start to respond only at the expected reward onset^96^. For this reason, virtually all head-fixed tasks fail to use timing metrics at all, even if in theory such metrics might be available^32,33,76–78^. Reaction times in our paradigm are based on a deliberate and consistent change in running direction. As such, they track the moment of target choice, rather than e.g. arrival at the target. The reaction times measured in this way can be as fast as 200 ms, the minimum delay one would expect from a visually driven motor response based solely on neuronal conduction delays (see Supplementary Fig. S2). This suggests that motor components do not add significant delays to our estimate of the moment of perceptual decision making. At the same time, the required motor response is metabolically costly enough to the animal that it only occurs due to a deliberate target choice, and random fluctuations in behaviour cannot be mistaken for a response. This is evidenced by the fact that the rate of false positives in the absence of a visible target is zero in our paradigm (see Supplementary Fig. S1). Having a reliable measure of decision timing opens up the option to e.g. analyse neuronal responses relative to each trial’s reaction time rather than stimulus onset, yielding a profile of action-related neuronal responses. Such analyses with reference to an animal’s internal timing can reveal crucial underlying processes that are not visible in analyses referenced to external timing^80,97–101^.

The primary performance metrics we extracted allow us to dissect cognitive processes contributing to performance, providing an unprecedented window into task processing and decision making in mice. For instance, by observing the conjunction of all primary metrics with the PR score, we were able to demonstrate that alertness fluctuates rhythmically. Mice have been known to exhibit spontaneous fluctuations of performance, yet most paradigms nevertheless simply pool performance over high- and low-attention phases. Others exclude trials based on cut-off points, e.g. when an animal’s performance falls below 80% of average performance and/or does not conform to expected psychophysical performance curves^102,103^. While trial exclusion can help to filter out low-performance phases that the researcher may not be interested in, it is difficult to avoid arbitrary cut-offs. Our paradigm classifies states of high and low alertness in a data-driven way, based on the bimodal distribution of local PR scores per animal. This can serve both to discard low-performance phases, and to find neural markers of attention. It also makes it possible to phenotype animals according to their scope for sustained attention (sATT score) by registering how much time they spend in high alertness.

Compared to classical measures of sustained attention (e.g.^40^), this approach yields several advantages: First, the task relies on spontaneously occurring behavioural dynamics, and as such does not require any specific cuing or additional task features. This makes training faster and more adaptable to different experimental designs, and dissociates the measurement of attentional capacity from the measurement of the ability to learn an attention task (see Supplementary Fig. S7). Second, by defining high and low performance individually for each animal, the sATT score is independent of global performance differences between animals, brought about e.g. by visual acuity (see Supplementary Fig. S7). Finally, the fact that rhythmic transitions between states of high and low alertness occurred consistently across virtually all animals suggests that this is a fundamental and intrinsic feature of attentive processing in mice. In contrast, behaviours quantified by classical rodent attention tasks like the 5CSRTT are more directly comparable to primate and human response schemes, but also require considerable training, suggesting that not only are they not intrinsic to mice, the underlying neuronal mechanisms studied in this way are also likely shaped by the task itself. Moreover, most attention tasks yield one overall measure of attention across an entire session, making it difficult to identify attention levels in individual trials with any level of accuracy. As we show here, attention fluctuates strongly and consistently throughout a session. Pooling these fluctuations into one average metric therefore misses out on some of the most salient and interesting features of the attentional process, and precludes direct links to ongoing neuronal activity. Note that the fluctuations of alertness on which the sATT score is based would be (and indeed were) barely noticeable based on global performance measures like hit rate - or even average reaction time. This further highlights the notion that behavioural metrics sensitive enough to identify ongoing behavioural states are a crucial step towards making sense of behavioural dynamics and their underlying neuronal circuit interactions^104^.

The fact that animals could be reliably cued using two different incentive schemes warrants particular emphasis. Cuing has rarely been attempted in mouse tasks, yet in this task both cuing schemes succeeded. This opens up important options to create mouse tasks that are more closely matched to paradigms studying cued attention e.g. in primates. This also demonstrates that mice are able to register, and adjust to, subtle reward contingencies with extreme precision. For instance, animals obviously detected the difference between a 10 μl reward for a >500 ms reaction time, and a 15 μl reward for a 400–500 ms reaction time, and increased their response speed accordingly (see also^105,106^). While we focused on temporal (i.e. trial-wise) cuing here, we expect that spatial cues (e.g. pointing out the likely position of the next target) would be at least as effective. These findings support the notion that mice possess cognitive abilities that are difficult to study, or even acknowledge, by simply translating behavioural assays from other species, but that are worth exploring on their own terms^104^.

Compared to other tests of goal-directed attention^3,10,12,37,38,40,42,44,48,60,72,94,107–110^, the cuing paradigm presented here can essentially be seen as a fast-learning distractor task with just one distractor (though more can easily be added). One point of difference to previous paradigms is that cued attention is not quantified based on absolute task performance in the presence of distractors, but based on the relative improvement of performance in cued trials, as captured by the cATT score. This procedure is designed to remove non-attention-related factors (e.g. visual acuity) that presumably contribute equally to baseline and cued performance. Most importantly, we measure performance improvements in cued trials via the CL index, a hybrid metric of response speed and accuracy, rather than a typical accuracy measure like hit rate. As a result, cued attention is quantified independently of whether an animal prioritizes speed or accuracy (see Fig. 6d), whereas in other paradigms animals that prioritize speed would be indistinguishable from inattentive animals.

Finally, it is important to note that sATT and cATT score are largely uncorrelated (see Fig. 6f). In human research, it is common practice to treat and measure different components of attention separately^1,3,8^. We provide, to our knowledge for the first time, a task for mice that measures two independent aspects of attention which can be directly translated to the concepts of attention research in humans. This means that these two attentional processes can be examined and linked within the same animal, rather than via two different tasks, presumably in different animals since mice do generally not learn multiple tasks very well.

In terms of visual neuroscience, our paradigm offers the unique possibility to obtain a full psychophysical curve of orientation discrimination in mice after less than a week of training. While we focus on stimulus orientation here, one could of course adapt the presented stimuli in order to measure e.g. contrast sensitivity. Psychophysical curves are one of the most basic and useful elements of vision research – yet they have only rarely been accomplished in mice. Most paradigms have measured visual discrimination in mice at a rather coarse level (e.g. using >45° orientation differences)^31,33,79,84^. The few paradigms to obtain a psychophysical curve required weeks to months of training, yet animals still seemed to drop out of training and/or achieve lower accuracy than in the current paradigm^32,74,77,78^. With the present task, we hope to make a fundamental tool of vision research more readily available to mice, creating the option of routinely testing advanced visual discrimination in mice.

What neuronal processes can be studied with this approach? One important application of our paradigm was already mentioned above: Precisely timed measurements of behaviour allow for the quantification of neuronal network activity based on internal rather than external timing (e.g. based on the moment of decision making rather than the moment of stimulus appearance)^80,98–101^. Second, we know that ongoing, ‘noisy’ neuronal dynamics have an important impact on visual processing^111–113^. To study the role of such non-repeating neuronal population activity further, it is important to relate it to its immediate perceptual and behavioural consequences on a moment-by-moment basis. Our task makes this possible by providing nuanced metrics of behaviour that can be tracked trial-by-trial rather than needing to be averaged into e.g. a hit rate before being related to neuronal activity. One field of research where such possibilities may be of particular interest is the study of Artificial Intelligence, which currently focuses strongly on reinforcement learning. In this context, the rich behavioural data our paradigm provides can help formulate Bayesian approaches to naturalistic reinforcement learning. Finally, the reason that sustained and cued attention are treated as separate processes in human research is not only that – like in our task – they are behaviourally independent. They also seem to involve largely distinct cortical circuits. Specifically, sustained attention seems to be supported by thalamus, locus coeruleus, frontal and parietal cortices, with neuromodulatory signaling by the norepinephrine system, while cued attention appears to rely on frontal eye fields as well as parietal lobes, supported by cholinergic signaling. Our task is ideally suited to dissect the interplay between these circuits, and to explore how population dynamics within and across these networks relate to attentive behaviour.

One potential concern with this task is that by relying on treadmill running, stimulus responses feature a large motor component. As a result, performance (e.g. reaction times) might depend on motor difficulty as much as on stimulus processing. We argue that this is largely not the case – at least not more than for any other rodent task. First, responses to the easiest stimulus conditions were close to the expected performance optimum (Supplementary Fig. S2), with hit rates clustering close to 1, and reaction times close to the expected minimum of 200 ms^114,115^. Thus, motor difficulty seemed to add negligible error variance when stimulus difficulty was low. The fact that mice learned to run on the treadmill within 5–10 minutes of first encountering it, and learned to steer to the left and right within 15–45 minutes of being presented with lateral targets, also suggests that they did not find the required motor response particularly difficult. Second, while responses like licking of lever presses seem more straightforward, and less affected by motor processing, they in fact contain a large ‘hidden’ motor component: The potential gain of reward far outweighs the energy investment of e.g. one lick. Since mice do not easily inhibit action under such circumstances, the act of refraining from licking in fact requires rigorous training^32,74,76,78^, and even so, responses cannot be guaranteed to be consistently stimulus-related. Touchscreen tasks on the other hand have the same potential issue as our task – since they require running towards a target, the contribution of motor activity is likely comparable, with the difference that free-running tasks cannot even measure motor outputs to the extent that virtual-environment tasks can, and in addition tend to give coarser estimates of performance markers like reaction times (see above).

In our view, the main limitation of the VEF task lies not in its response scheme, but in the nature of the visual stimulation it provides: Since the task is based on navigation in a virtual environment, the animal is causing its visual surroundings to move almost constantly. As a result, stimuli will continuously vary in size, spatial frequency, retinotopic location etc. Thus, this task is not designed for precise, receptive-field-specific presentation of visual stimuli. As a consequence, neuronal responses recorded over the course of the task are more complex and less tightly controlled than in other paradigms e.g.^44,74,77,79^. Not only will neuronal activity vary with changing stimulus properties, it will also be modulated by e.g. visual flow, locomotion^75,116–120^ and arousal^76,121–123^. Therefore, unlike in more controlled paradigms, e.g. those measuring receptive-field-specific effects of attention in primates^107–110^, neuronal activity in this context will continuously integrate and represent multiple variables related to the visual world, making it more complicated to isolate the effect of attention on individual neuronal responses.

To some extent, this point can be addressed by e.g. limiting stimulus presentation to a short time (subsequently letting animals steer towards grey target walls), by projecting the virtual environment on regular computer screens rather than a spherical dome to simplify retinotopic mapping, or by transitioning from running to more passive responses (e.g. licking). However, overall the approach presented here is simply not designed for such aims. Instead, it is suited to track how visual information and cognitive factors are integrated globally in a naturalistic context, and converted into behavioural responses. This will make it more challenging to extract individual behaviourally relevant features of neuronal activity. Yet it is also a more realistic representation of attentive processing in a natural context, and is therefore in our opinion more likely to highlight robust principles of neuronal population coding beyond the confines of a laboratory setup. In a natural environment, movement and visual stimulation, as well as top-down modulation (e.g. by alertness), interact continuously. While well-defined visual stimuli appearing on a clutter-free background have been central in mapping neuronal circuits, behaviourally relevant processing is unlikely to work this way. For instance, recent studies indicate that even the simple act of running modifies sensory responses dramatically compared to those encountered during passive viewing^97,117,119,120,124–126^. This task aims to provide a tool to more precisely disentangle the complex and messy neuronal interactions that the brain generates throughout ongoing attentive behaviour.

## Methods

Data were collected from 12 male wild-type mice (Strain: C57-BL6, Charles River). All animal procedures were approved by the Ethical Committee on Animal Experimentation of Radboud University Nijmegen (RU-DEC) and the Dutch Ethical Committee on Animal Experimentation, and in accordance with the EU guidelines for animal experimentation.

### Behavioural setup

Mice were head-fixed atop a floating-ball treadmill consisting of a styrofoam ball (Graham Sweet Studios; Cardiff, UK; diameter 20 cm) floating on air in a custom-made mold (University College London workshops). The treadmill was surrounded by a spherical screen (Fibresports UK; Basildon, UK; diameter 136 cm) covering 270° of visual angle. A virtual environment was projected onto this screen using a projector (Optoma X501; Optoma; Fremont, US) positioned behind the screen and a spherical mirror located underneath the treadmill (diameter 38 cm; see Fig. 1a,b for a schematic and photo of the setup). Mice were head-fixed by attaching two holders with fixation screws to the hinges of an implanted head-plate (designed by Jasper Poort, University College London; all components supporting the dome, treadmill and head holder were made by Thorlabs; Dachau/Munich, Germany).

To capture locomotion, two computer mice (Logitech G500; Newark, US) were placed along the horizontal axis of the treadmill - one behind the animal, and one to its right, forming a 90° angle - to register forward and lateral movement of the treadmill, respectively. Readouts of the ball movement were retrieved at a frequency of 60 Hz using custom Python scripts integrated in the virtual environment (programmed in Blender; www.blender.org) and adapted from the Gnoom platform by Christoph Schmidt-Hieber (https://research.pasteur.fr/en/software/gnoom/).

Liquid reward was delivered through a tube placed on a small metal holder in front of the animal (built in-house). The tube was opened and closed by a pinch valve (NResearch Inc.) driven by TTL pulses from an Arduino Duemilanove board which was connected to the virtual environment. A lick sensor (built in-house) was integrated in the tube holder, and provided an analogue measurement of licking activity, which was recorded with a sampling rate of 60 Hz via a second Arduino Duemilanove board, and stored together with the locomotion traces. The lick sensor itself was based on simple circuit closing: A ground wire was connected to the animal via one of the head holders. At the same time, the metal holder containing the reward tube provided an analogue input to a second Arduino Duemilanove board. Whenever the animal made a connection to the reward tube, e.g. by touching the outside of the tube, or the reward liquid, the circuit was closed, sending the recorded signal sharply to zero. Unlike movement/vibration or beam-breaking sensors, this sensor did not need to be calibrated and did not miss licks even when mechanical movement was minimal.

### Task structure

While different stages of training differed in specifics like target position (see below), a training session generally adhered to the following structure: One hour before the session, mice were water-deprived. They were then head-fixed on the floating-ball treadmill and faced with a series of progressively more complex tasks, navigating a virtual environment based on visual cues. To succeed in a trial, animals had to run through a wall displaying the target grating. There were two different types of miss trials: Animals could either not run through any wall, or in advanced training stages (5–7), they could run through the wall displaying a distractor grating. In other words, this was not a forced-choice paradigm.

When a trial was completed successfully, the animal would immediately hear a ‘reward’ tone and receive a liquid reward dispensed from the tube in front of its mouth. A single reward consisted of 10 μl of sugared soy milk. In the final task, cued trials (20% of trials) would be announced by an auditory signal, and if successful, animals would receive 2–4 rewards in that trial, i.e. 20–40 μl of reward liquid. In case of a failed trial, animals would encounter a ‘time-out’ corridor – a dark corridor they had to traverse in order to initiate the next trial. This corridor could have two different lengths, corresponding to small and large punishment. If animals in later training stages not only missed the target, but also navigated towards the distractor, they would additionally hear a ‘punishment’ tone of loud white noise. After completing the ‘time-out corridor’, animals would re-initiate the previous trial until they succeeded. If animals restarted the same trial more than once, they would generally receive gentle manual guidance towards the correct target. Note that repeated trials were not analysed since the animal’s performance depended on the previous trial (for example, some animals learned to automatically move to the opposite side after a miss trial) as well as on the manual guidance from the experimenter.

Animals were allowed to perform the task until either the number of licks or the number of correctly initiated trials dropped, signalling fatigue. After training, animals received a performance-dependent ‘bonus’ reward (a piece of peanut or raisin). They were then given play time with litter mates that were being trained on the same day, and received dry food in their home cage at least 30 minutes after training had concluded.

The task was structured as follows: Animals were initially presented with a grey target wall located in the centre of the virtual environment. Once the animal moved towards the target, it would cross an invisible trigger threshold, causing the target to move either to the left (40% of trials), centre (20%) or right (40%), and display a circular sinusoidal grating. Centre trials simply required the animal to keep running straight ahead and were not analysed further. When targets moved to the side, a distractor target simultaneously moved to the contralateral location and displayed a competing grating of different orientation. Targets displayed gratings oriented more horizontally, while distractors displayed gratings oriented more vertically. The easiest discrimination trials thus featured a horizontal target and vertical distractor (90**°** orientation difference), while the hardest discrimination featured a 42.5**°** target and a 47.5**°** distractor (5**°** difference, see inset in Fig. 2c). A schematic of the task is shown in Fig. 2c.

### Training protocol

For surgical procedures and the pre-training handling protocol, please see Supplementary Methods. Mice were housed individually on a reversed light cycle (Lights off from 8 am to 8 pm), and were trained from ~2–6 pm. We usually trained 2–4 animals per day, in the same sequence, so that each animal had a fixed training time. Every animal completed one training session per day, which typically lasted ~45–60 minutes (minimally 20 minutes, maximally 90 minutes). A session was terminated before 60 minutes if a mouse stopped licking or running, failed to initiate trials or stopped approaching targets. A session was extended beyond 60 minutes if an animal was still licking for reward and approaching targets efficiently, and if a new training stage had been introduced shortly before. Depending on their performance, animals could progress through up to three training stages per session (see next paragraph).

The behavioural training leading up to the discrimination task consisted of seven consecutive stages, (see Havenith *et al.* (under review)). Briefly, the training steps were:

(A) In the first training stage, animals were placed on the floating-ball treadmill in the dark and rewarded every time they moved forwards by a couple of steps.
  (B) When animals stopped showing signs of stress and were able to move forwards they were moved to next training stage, featuring low-contrast target gratings in a dimly lit corridor. Targets always displayed a horizontal sinusoidal grating to associate horizontal orientation with reward. Target walls filled the entire corridor, making it impossible for animals to avoid them. Animals were rewarded whenever they moved through a target wall. When a mouse learned to walk independently and lick for reward, the second training stage began.
In the second training stage, the corridor surrounding the target walls was removed. Opening up the virtual space allows animals to miss the target, and thereby forego reward. After a few seconds of running, the next target wall would then appear. In this way, animals learned to navigate actively towards the target. When animals had a success rate > = 80%, they advanced to training stage 3.
In training stage 3, targets initially appear at the centre of the environment, then move at a 45° angle to the left or right when the animal crosses an invisible trigger threshold in front of the initial target location. If animals did not approach the centre target, the trial was reset. Thus, the animal initiates the trial by running towards the centre target. Animals tended to immediately attempt to follow targets, suggesting that they already treated the environment as a regular space, and targets as approachable objects. Therefore, step 3 mainly served to train steering skills. When animals achieved a success rate of > = 80% for both target locations, they were transferred to training stage 4.
In training stage 4, targets were initially located in the centre as in stage 3, but could now move to the left (40% of trials), right (40% of trials) as well as backwards (20% of trials). Trials were pseudo-randomized, evening out across chunks of 10 consecutive trials. A ‘weak’ distractor in the form of a low-contrast vertical grating moving in the opposite direction to the target appeared in trials when the target was moving to the left or right. When animals chose to navigate through the distractor, they experienced an auditory white noise stimulus followed a ‘time-out’ corridor. It was crucial to move on from this training stage as soon as animals reached a > = 80% success rate in order to avoid associating reward with stimulus contrast rather than orientation.
Training step 5 was the same as training step 4, except that distractors now displayed vertical sinusoidal gratings at the same contrast as the targets. In addition, to test the effect of attentional cuing (see Fig. 6), at this point we added a cuing tone in 20% of trials which signalled that if the animal responded correctly it would receive increased reward (multiple drops of soymilk), whereas if the animal responded incorrectly it would face increased punishment (a longer punishment tone and longer time-out corridor).
In the final training step, trials with progressively decreasing orientation difference between target and distractor were introduced gradually. While the original target and distractor had an orientation of 0° and 90°, respectively, in subsequently added trials the orientation of both target and distractor approached 45°. As a result, a maximum of eight orientation differences was randomly interleaved in a training session: 90° (target 0°; distractor 90°); 70° (target 10°; distractor 80°); 50° (target 20°; distractor 70°); 30° (target 30°; distractor 60°); 20° (target 35°; distractor 55°); 15° (target 37.5°; distractor 52.5°); 10° (target 40°; distractor 50°); and 5° (target 42.5°; distractor 47.5°). Each trial difficulty was added when animals had adapted to the previous one such that their success rate in that difficulty was > = 70%. Not all animals reached the most difficult task conditions – for some animals, performance decreased steeply when trials with 5° or 10° orientation differences were added. In those cases, we stopped adding more difficult trials.

Note that in training stages 4–6, centre trials did not call for a target choice, and were not analysed further. Rather, they served training purposes: They provided a baseline of trials in which animals were highly likely to be rewarded, heightening motivation; and they prevented animals from slowing down before every target shift in anticipation of having to change direction.

### Data collection

Behavioural data were recorded at a sampling rate of 60 Hz using custom Python scripts integrated in the virtual environment (programmed in Blender; www.blender.org) and adapted from the Gnoom platform provided by Christoph Schmidt-Hieber (https://research.pasteur.fr/en/software/gnoom/). The analogue read-outs of forward and lateral movement were translated into the locomotion within the virtual environment while also being recorded in a text file. The corresponding lateral (X) and longitudinal (Y) position of the animal within the virtual environment were recorded at the same time. In addition, a lick sensor (see above) provided another analogue input via an Arduino Duemilanove board (Arduino; Somerville, US), which was read and stored together with the other readouts.

The translation factor of actual locomotion to movement within the virtual environment was 3.0, i.e. animals ran three times the distance they traversed in the virtual environment. The reason was that mice actually ran so fast on the treadmill that once targets came close enough to be perceived at all, animals would already have run past the target before they could even begin to change running direction.

Data were stored in two separate text files: One contained the time stamps of discrete events per trial generated within the game (trial onset, trial offset, time of target shift), as well as some simple behavioural variables per trial (target reached or missed, amount of reward received). The second file contained a continuous 60-Hz read-out of behavioural measurements, specifically locomotion and licking behaviour. Finally, training sessions were regularly filmed with a small webcam (Logitech C310; Logitech; Newark, US) positioned at the right-hand corner of the virtual environment dome.

### Primary single-trial performance metrics

Data were analysed using custom scripts written in Matlab (Mathworks; Natick, US). First, the recordings of locomotion, virtual position and licking were cut into trials based on trial onset and offset, and then analysed to yield seven primary behavioural metrics per trial. The primary performance measures shown in the main text were defined and measured as follows:

1. Hit index
  The correctness of a trial was classified as 1 when the animal touched the target, −1 when it touched the distractor, and 0 when it touched neither. Since this measure is similar but not identical to the classical hit rate, we refer to it as hit index.
2. Target Distance
  The target distance is a continuous measure of accuracy, and is defined as the lateral distance between the animal and the target at the end of the trial (‘finish line’). It was computed in the following way:
  1 $$TD=\,|\frac{{X}_{T}-\,{X}_{M}}{{X}_{T}}|$$
  where TD is the target distance, X_T_ is the lateral (X) position of the target edge closest to the animal, X_M_ is the lateral (X) position of the animal at the longitudinal (Y) level of the target, and ΔX_T_ is the distance between two adjacent target positions.
  The distance between the animal and the target is normalized by the distance between two adjacent target positions (ΔX_T_). This is done to give an intuitive interpretation of the resulting values and make them comparable across different task implementations: Irrespective of the specific spatial layout of the task, a target distance of 1 always means that by the end of the trial, the animal was so far removed from the target that it could have touched a different target position altogether. For example, if the target was positioned on the left and the animal ran straight ahead, it would result in a target distance close to 1, whereas if the animal steered to the right, it would generate a target distance close to 2. Note that all correct trials by definition result in a target distance of 0.
3. Path reliability (PR) score
  The PR score is a third, complementary, measure of accuracy. It assesses the spatial precision with which animals aim for the target positions. As such, it is computed by comparing running paths across multiple trials (see Fig. 3c). To assess the spatial replicability of running paths regardless of their time course, we first created a standardized representation of each running path: For Y positions starting at the location of the target shift trigger and moving in steps of 2 cm up to the target position, we computed the average lateral (X) position of each path. For each point in Y, we then computed the discriminability between the average X positions belonging to running paths for the left and right target trials. To do so, we used Cohen’s D^127^, a standard measure of discriminability which normalizes the difference between two averages by the corresponding pooled standard deviations:
  2 $${D}_{y}=\frac{|X{L}_{y}-X{R}_{y}|}{\sqrt{\frac{({n}_{L}-1)\cdot {s}_{L,y}^{2}+({n}_{R}-1)\cdot {s}_{R,y}^{2}\,}{{n}_{L}+{n}_{R}-2}}}$$
  where D_y_ is the discriminability at longitudinal position Y, XL_y_ is the mean X position at position Y across all running paths for which the target is located on the left, XR_y_ is the same for targets located on the right, n_L_ is the number of trials with targets on the left, n_R_ is the number of trials with targets on the right, s_L,y_ is the standard deviation of all X positions for position Y with the target on the left, and s_R,y_ is the same for targets on the right. This yielded a vector of discriminability values across Y space, as shown in Fig. 3c. The maximum of this vector (e.g. 4.0 in Fig. 3c) was used to represent the Path Reliability of a group of trials.
  We applied the PR score as a global measure across all trials in a session, as a stimulus-dependent measure across the trials of each stimulus difficulty (see Fig. 4a,b), and as a local running average per trial. In the latter case, for each trial we computed the local Path Reliability by taking into account a total of 15 trials, i.e. 7 trials prior to and 7 trials following the trial in question. For the first and last 7 trials of a session, local Path Reliability was not computed. Such running averages of local Path Reliability are shown in Figs 4a and S1, as well as being used for all classifications of High-Alert and Low-Alert trials (Figs 5, 6d,f, S3–S5).
  Path Reliability decreases with incorrect trials (since incorrect paths increase the standard deviation of X positions) and increases when paths leading to the same target are not only correct but also spatially uniform (i.e. replicable), decreasing the standard deviation. Thus, given the spatial layout of this task (distance between left and right targets: 24 cm; target width: 9 cm, see Fig. 1c), a set of correct but spatially variable paths would yield a Cohen’s D of up to ~4.5. Values above this, as seen e.g. in Figs 5a and S1, signify that running paths were not only predominantly correct, but also spatially replicable beyond merely hitting the target. Note that centre trials were not included in this measure since they did not require a behavioural choice and were rather used as reset trials in which animals could gain reward simply by continuing to run.
4. Path surplus
  The path surplus is a metric assessing whether animals take the shortest route towards the target or whether they make additional direction changes. As such, the path surplus decreases with the animal’s skill in moving on the treadmill, but more importantly, it increases when an animal ‘changes its mind’ and changes running directions midway to a target location. To compute the path surplus, the length of the animal’s running path from the point of target choice (measured through the reaction time) to the target location is compared with an ideal path length. The ideal path length is computed as the Euclidean distance between the animal’s position at the point of target choice, and the target position:
  3 $${L}_{i}=\sqrt{{({X}_{T}-{X}_{RT})}^{2}+{({Y}_{T}-{Y}_{RT})}^{2}}$$
  where L_i_ is the ideal path length, X_T_ is the position of the target along the X axis, Y_T_ is the position of the target along the Y axis, X_RT_ is the X position of the animal at the point of the reaction time, and Y_RT_ is the equivalent in Y. The actual path length was then computed as follows:
  4 $${L}_{R}={\sum }_{t=\,1}^{t=\,n-1}\sqrt{{({x}_{t+1}-{x}_{t})}^{2}+{({y}_{t+1}-{y}_{t})}^{2}\,}$$
  where L_R_ is the actual path length, t denotes all consecutive measurements of x and y locations, beginning from the point of the target choice (t = 1) until one data point before the trial end (t = n − 1). Finally, the path surplus PS was computed as:
  5 $$PS=\frac{{L}_{R}}{{L}_{I}}-1$$
  Thus, a path surplus of 0 would mean that the actual path length was equivalent to the ideal path length, while a path surplus of 0.5 would indicate that the actual path length was 50% longer than the most efficient path length. Note that a path surplus <0 could occasionally occur because the ideal path length was computed using the lateral position of the target centre, whereas animals might hit the closest edge of the target, shortening the path slightly compared to the ‘ideal path’. Note that the path surplus does not take into account the correctness of the trial (since this was already addressed by target distance, hit index, and PR score) – it only reflects how efficiently animals move from the point of target choice towards the target location they reach (or are closest to) at the end of the trial. In other words, we took into account the target location the animal presumably chose, rather than the target location that is correct.
5. Reaction time
  For all trials in which targets moved laterally, we computed a reaction time based on the change in running direction that the animal exhibited after the target had shifted location. The computation was based on the running paths recorded from 0.5 seconds before the target shift to 3 seconds after the target shift, with the aim of pinpointing the moment of largest change in running direction.
  Naturally, a local running direction for a specific time point t cannot be defined by that time point alone, but needs to take into account the animal’s displacement throughout a time window surrounding that time point. We define this time window as τ:
  6 $$\tau =[t-T,t+T]$$
  where t is a time point (i.e. sampling point of the recording) and T is a fixed interval determining the size of the averaging window. Note that longer time windows will filter out ‘noise’ in the running trajectory (e.g. based on a particular step the animal made) but will also ‘gloss over’ sudden changes in running direction. We therefore initially computed estimates of reaction times based on T values of 2, 5, 10, 15, and 25 sampling points, corresponding to 33, 83, 167, 250 and 417 ms (given a 60 Hz acquisition rate). We found that T = 10 (i.e. a window of 20 sampling points, or 333 ms) yielded robust estimates that also best represented the converging results of all other parameter constellations.
  In the next step, one option would be to use τ as a sliding window to determine the average running direction at each point of the running trajectory, and then compare the estimated running directions in order to find the largest deviation. To do so, each part of the running trajectory would first have to be fitted with a linear function, and the resulting direction estimates would then be compared. However, to arrive at a robust estimate of direction changes given the small amount of data available in each trial (3.5 seconds of running trajectory * 60 Hz sampling frequency = 210 samples per trial), we were keen to minimize the number of parameters fitted to the running trajectories. We therefore directly computed a linear regression between two partially overlapping portions x_τ_ and x_τ+Δ_ of the animal’s running path, halving the number of fitted parameters:
  7 $${x}_{\tau +{\rm{\Delta }}}={b}_{t}\cdot \,{x}_{\tau }+{m}_{t}+{{\epsilon }}_{t}$$
  where x_τ_ is an animal’s lateral (X) positions over time window τ, x_τ+Δ_ is a corresponding vector of X positions, but shifted forwards in time by Δ; b_t_ is the slope of the function converting x_τ_ to x_τ+Δ_, m_t_ is the offset of the function, and ε_t_ is the corresponding error term. The linear regression function was fitted in Matlab using the criterion of least square errors, minimizing ε^2^. After exploring Δ values (i.e. time shifts) of 1 to 15 sampling points (i.e. 16 to 250 ms), we found that a Δ of 5 sampling points, i.e. 83 ms (or 25% of the 20-sampling-point window we chose as the length of each X vector) worked well to identify the biggest changes in running direction in a way that corresponded to ‘by-eye’ estimates.
  Based on these fitted functions, we disregarded m_t_ - since an offset would simply signal a lateral displacement of x_τ+Δ_ relative to x_τ_, which would be expected if the animal is moving. Instead, we focused on the slope b_t_ of the regression function. If b_t_ at time point t was close to 1, it indicated that the animal was showing the same lateral movement in both portions of the running path, i.e. running in the same direction. In contrast, slopes higher or lower than 1 would indicate changes in running direction. As a result, the change in running direction could directly be estimated without first estimating the running direction of each path portion separately. For the vector of slope estimates b_t_ across all time points t (see Fig. 3b), we determined the reaction time as the point at which the slope was most different from 1, limiting the range of possible reaction times to 0.1–1.25 seconds. Reaction times <0.1 seconds were physiologically unlikely, and reaction times >1.25 seconds indicated trials in which the animal was not responding to the stimulus to the best of its ability and/or not perceiving the target stimulus for some reason. If b_t_ differed from 1 by <0.1, this indicated that the animal had not substantially changed its running direction (e.g. because it was already running to one side before the target moved). In those trials reaction times were not defined.
6. Lick position
  To assess whether animals were licking more in anticipation of or in response to reward, we quantified which longitudinal (Y) location animals were in when licking (see e.g. Figs 3b and S1). For each trial, we took into account the Y position of the target ±30 cm. Since the space between the animal’s starting position and the target had a length of 62 cm, this means that we considered approximately the last half of the virtual Y space in the trial leading up to the target, and the first half of the following trial’s Y space. The average lick position per trial was then computed as the mean distance of the included lick positions from the target’s position in Y.
7. Running speed

The mean running speed for a trial was computed by averaging the running speeds starting at the target shift up to point when the animal was within 10 cm of the target. We used this analysis window to ensure a good representation of the animal’s response speed: Before the target shift, running speed tended to be dictated by the previous trial. For example, when animals were still licking for reward from the previous trial, they would start out running more slowly irrespective of the current trial. On the other hand, some animals tended to slow down close to the target to start licking for reward. In our view, including these portions of the trial would yield a less accurate representation of the speed with which the animal approached the target.

### Secondary performance metrics

Based on these primary performance indicators, we also carried out secondary analyses, listed here in the order of appearance in the main text.

1. Visual discrimination thresholds (Figs 4, 5e,f and 6a,b)
  To identify each animal’s perceptual threshold for orientation differences, we used the psychometric curves of average hit index, target distance and PR score as a function of ΔOri (Fig. 4a). Using the ‘fit’ function in Matlab, we fitted the psychophysical curves with a logistic function:
  8 $$f({\rm{\Delta }}\mathrm{Ori}\,)=\frac{L}{1+{e}^{-s\ast ({\rm{\Delta }}\mathrm{Ori}-o)}}+\varepsilon $$
  where f(ΔOri) is the observed psychophysical curve f as a function of the orientation difference ΔOri, L is the maximum value of the curve, s is the steepness of the curve, o is the horizontal offset of the curve, and ε is the error term between the sigmoid function and the observed psychophysical curve.
  From the resulting logistic functions, a discrimination threshold was defined as the ΔOri at which the function reached the criterion value. For the hit index, the criterion was 0.2 (with 1 representing perfect performance and 0 representing chance); For target distance, the criterion was 0.82 (with 0 representing perfect performance, and 1 representing chance); For the PR score, the criterion was 1.25 (with scores >3 representing correct performance, and scores <1 representing chance). In theory, the criterion values would be easiest to determine by having animals repeatedly complete trials with a ΔOri of zero, and measuring the resulting performance. However, such catch trials quickly impact overall performance across all ΔOri as animals get confused and demotivated (data not shown). We therefore chose to determine criterion values using a bootstrapping procedure based on the variability of animals’ responses to true orientation differences (ΔOri > 0). Bootstrapping was done as follows: In order to decide whether a particular outcome (e.g. a hit index of 0.27) represented chance or above-chance visual discrimination, we needed to estimate the error variance associated with such an outcome. Since psychometric curves consist of the average performance per ΔOri, the relevant measure of error variance is the standard error of the mean (SEM). To estimate the SEM for hit index, target distance and PR score, respectively, we relied on a bootstrapping procedure: For each ΔOri, we repeatedly sampled 20, 40, 60, and 80% of trials and computed the resulting SEM as SD/√n, where n is the number of trials (10 bootstrap repetitions each per ΔOri and trial fraction). This procedure was repeated for each animal, and all resulting SEMs were pooled. We then used the median of the pooled SEM distribution as the criterion threshold for non-random performance. We chose to use the median of the SEM distribution (rather than e.g. the 95^th^ percentile) because a perceptual threshold is generally taken to reflect the point at which there is a 50% probability of correct performance. The median SEM should represent that case closely – given that SEMs were bootstrapped by sub-sampling trial numbers, performance at the criterion value should be significantly above chance in more than 50% of tests. For the hit index, the median SEM was 0.19, leading to a criterion of 0.2; For target distance, the median SEM was 0.17, leading to a criterion of 0.82; For the PR score the median SEM was 1.20, leading to a criterion of 1.25.
2. Establishing High- and Low-Alert trials: Assessing the bimodal distribution of local PR scores (Figs 5, 6d,f, and Supplementary Figs S3–S5)
  To determine whether PR scores were distributed according to a bimodal distribution, we computed the bimodality coefficient as suggested by^93^:
  9 $$BC=\frac{ske{w}^{2}+1}{kurt-3+3\cdot \frac{{(n-1)}^{2}}{(n-2)\cdot (n-3)}}$$
  where BC is the bimodality coefficient, skew is the skewness (third moment) of the distribution, kurt is the kurtosis (fourth moment), and n is the number of samples. While commonly used, this coefficient does not provide a statistical test, but gives a critical value of BC = 0.55, above which distributions can be assumed to be bimodal rather than unimodal (see^93^). For example, a bimodal distribution consisting of two overlapping normal distributions would receive a bimodality coefficient >0.55 once the distance between the two modes exceeds ~3.6 times the standard deviation. The resulting classification was also largely consistent with the classification one would make by eye.
  When a distribution had been classified as bimodal, we next identified the cut-off point between the two modes of the distribution, using the approach suggested by^128^. This analysis simply compares the amount of variance in two portions of a (presumably bimodal) distribution with the total variance across the entire distribution:
  10 $$F=\frac{Va{r}_{All}}{mean(Va{r}_{part1},Va{r}_{part2})}$$
  where Var_All_ is the total variance across the distribution and Var_part1_ and Var_Part2_ are the variances in the parts of the distribution above and below the cut-off point, respectively. The distribution is repeatedly cut in two at different points, and the cut-off point between the two modes is then determined as the one that results in the largest F value (see Fig. 5a). High-Alert trials were subsequently defined as trials whose local PR score exceeded the individually determined cut-off criterion of an animal’s particular distribution of PR scores; and Low-Alert trials were defined as trials whose local PR score remained below the cut-off criterion (see SupplementaryFig. S4).
3. sATT score (Figs 5d, 6f)
  The sATT score was computed as the fraction of time spent in High-Alert trials, compared to the overall time spent in all trials. As such, we first computed the durations (from trial onset to trial offset) of all trials an animal completed, then computed the sATT score as the sum of High-Alert trial durations, divided by the sum of all trial durations.
4. Cognitive Load Index (Fig. 6d, Supplementary Fig. S5)
  The Cognitive Load (CL) index is a composite measure combining response speed and response accuracy in order to make overall task performance comparable across animals, sessions and trials regardless of whether speed or accuracy was prioritized. The CL index linearly combines three measures defined above: Reaction time, target distance and path efficiency. To this end, all three measures were first normalized in order to be able to sum them linearly regardless of scale. This was done by scaling the value for each trial relatively to the overall minimum and maximum of the measure in question:
  11 $${n}_{tr}=\frac{{m}_{tr}-{M}_{5}}{{M}_{95}-{M}_{5}}$$
  where n_tr_ is the normalized measure for trial tr, m is the original measure for trial tr, M_5_ is the value of the 5^th^ percentile of the measure’s overall distribution across all recordings (representing the minimum of the measure excepting potential outliers), and M_95_ is the corresponding 95^th^ percentile (representing the maximum excepting potential outliers). Thus the majority of normalized values would fall into the range between 0 to 1. To arrive at the CL index for each trial, the three measures would then be combined as follows:
  12 $$C{L}_{tr}=0.5\cdot N{R}_{tr}+0.25\cdot N{T}_{tr}+0.25\cdot N{P}_{tr}$$
  where CL_tr_ is the CL index for trial tr, NR_tr_ is the normalized reaction time, NT_tr_ is the normalized target distance and NP_tr_ is the normalized path efficiency.
  The resulting CL index generally took values between 0 and 1, with an overall range of 0 to 3.4 (Mean ± St.Dev.: 0.39±0.25). The reason for the specific choice of weights used to sum reaction times, target distance and path efficiency was that we aimed for speed and accuracy to be represented in equal measure. As such, the weighting of the three components reflects the fact that reaction time is the only measure of response speed, while target distance and path surplus are complementary measures of response accuracy. This approach seemed to result in a balanced representation of speed and accuracy, as evidenced by the effects of speed and accuracy cuing on the CL index, as shown in Fig. 6d.
5. cATT score (Fig. 6e,f)
  The cATT score is a representation of the extent to which animals improved their performance in cued trials. Since we aimed to assess performance changes irrespective of performance style (prioritizing response speed or accuracy), this metric was based on the CL index. Specifically, it was computed by normalizing the change in CL index for cued versus non-cued trials, normalized by the average CL index across all trials:
  13 $$cATT=\frac{C{L}_{NC}-C{L}_{C}}{C{L}_{All}}$$
  where CL_NC_ is the average CL index in non-cued trials, CL_C_ is the average CL index in cued trials, and CL_All_ is the average CL index across all trials.
6. Error prediction (EP) index (results presented in detail in Havenith *et al.* (under review))

The EP index is based on the normalized difference between reaction times, path surplus and lick position in hit trials versus miss trials. The normalized difference in reaction times was computed as follows:

14 $${\rm{\Delta }}RT=\frac{R{T}_{Miss}-R{T}_{Hit}}{R{T}_{Miss}+R{T}_{Hit}}$$

where ΔRT is the normalized difference, RT_Hit_ is the average reaction time in hit trials, and RT_Miss_ is the average reaction time in miss trials. Note that ΔRT varies between −1 and 1, and takes on positive values when animals react slower in miss than in hit trials (i.e. correct prediction of trial outcome). The normalized differences in path surplus and lick location were computed in the same way. The error prediction index was then generated as a simple average of the three measures:

15 $$EP=({\rm{\Delta }}RT+{\rm{\Delta }}PS+{\rm{\Delta }}LL)/3$$

where EP is the error prediction index, ΔRT is the normalized difference in reaction times, ΔPS is the normalized difference in path surplus and ΔLL is the normalized difference in lick locations. The RP can therefore take on values between −1 and 1, with positive values indicating correct reward prediction.

## Statistical tests

Estimated statistical power and data structure for all statistical tests used in this study are summarized in Supplementary Table S1.

### Statistical significance of correlation coefficients

The statistical significance of individual correlation coefficients (see Figs 4b, 5f,g, 6b,c, 6f, S3 and S5) was directly extracted from Matlab’s ‘corrcoef’ function. To test the statistical significance of groups of correlation coefficients (see Fig. 5b, S3 and S6), we applied a simple t-test evaluating the sample’s difference from zero (see below for correction for multiple comparisons).

### Correction for multiple comparisons (Fig. 5b, S3, S6)

In cases when multiple tests were applied across several performance measures (e.g. Fig. 5b), we evaluated the resulting p-values against critical α values produced by the Dunn-Sidak Correction for multiple comparisons^129^:

16 $${\alpha }_{Corr}=1-{(1-\alpha )}^{\frac{1}{n}}$$

where α_Corr_ is the corrected critical error probability based on the desired family-wise error probability α, and n is the number of independent comparisons. In our case, we computed two α_Corr_ corresponding to α = 0.05 and 0.01 (indicated with 1 and 2 stars, respectively, above Figs 5b, S3b and S6a). For Figs 5b and S3b, the number of comparisons was 5 (6 performance measures, of which hit index and target distance are mathematically dependent, leading to a maximum of 5 independent comparisons). For Fig. S6a, the correction was across 3 comparisons (4 metrics, with the same dependence between hit index and target distance).

## Electronic supplementary material

Supplementary Materials

## Data Availability

The data sets and analysis tools presented in the current manuscript are available from the corresponding author on reasonable request.
