# Supplementary material for: The Virtual-Environment-Foraging Task enables rapid training and single-trial metrics of attention in head-fixed mice
Source: Sci Rep. 2018 Nov 26;8:17371. doi: 10.1038/s41598-018-34966-8 (PMC6255915; doi:10.1038/s41598-018-34966-8)
Supplement: Supplementary file 1 — Supplementary Materials [file 41598_2018_34966_MOESM1_ESM.pdf]

Supplementary Materials

The Virtual-Environment-Foraging Task enables rapid training and single-trial metrics of attention in head-fixed mice

Martha N. Havenith, Peter M. Zijderveld, Sabrina van Heukelum, Shaghayegh Abghari, Jeffrey C. Glennon, Paul Tiesinga

Contents

1) Supplementary Methods
2) Supplementary Note 1: Addressing confounds in attention tasks for mice
3) Supplementary References
4) Supplementary Figure S1: Primary performance metrics for one example session
5) Supplementary Figure S2: Mice perform consistently close to the limit of their ability
6) Supplementary Figure S3: The PR score mirrors shifts between high and low alertness
7) Supplementary Figure S4: Distributions of High- and Low-Alert states in four example sessions
8) Supplementary Figure S5: High-Alert states and Cued-Attention states improve performance independently but to similar extent - particularly for difficult stimuli
9) Supplementary Figure S6: Speed-accuracy trade-off is reduced by task expertise, alertness and cued attention
10) Supplementary Figure S7: sATT score and cATT score can be dissociated from visual acuity and learning ability
11) Supplementary Table S1: Summary of statistical tests

Supplementary Methods

Surgical procedures

To implant the head-plate needed for head fixation, anaesthesia was induced using isoflurane, and maintained with a mixture of Ketamine (Eurovet Animal Health; Bladel, Netherlands; 75 mg/kg), Dextomidor (Zoetis; Parsippany, US; 0.5 mg/kg) and saline (0.4 ml/kg), injected intraperitoneally (2.1 ml/kg). A circular aluminum head-plate (0.2g) was then implanted in the following steps: A circular portion of skin was removed on top of the skull, and the surrounding skin was glued to the skull with small amounts of superglue. The skull was then cleaned of tissue using a scalpel blade, and the head-plate was attached using Superbond (Sun Medical Co., Ltd; Moriyama, Japan). General analgesia during and after surgery was ensured by adding Rimadyl (Carpofen 5% with Ethanol 10%; Pfizer; New York, US; 60 mg/l) to the drinking water 2 days prior to surgery until 5 days post-surgery. In addition, local analgesia during the surgery was provided by treating exposed skin with lidocaine cream (EMLA). After surgery, animals were injected with Antisedan (Zoetis; Parsippany, US), diluted in saline 1:10 (5 mg/kg).

Pre-training protocol

Pre-training and training protocols are described in detail in [1]. Briefly, before head-plate implantation, mice were housed in groups in an enriched environment and had free access to dry food and water. After head-plate implantation, mice transitioned to individual housing with a reversed 12-12 day-night cycle. Mice were allowed at least one week of recovery, with intermittent contact with littermates. 3-5 days prior to training, animals were put on a restricted food schedule (2.0 - 2.5g of dry food/day) designed to reduce their body weight by 20%. When they reached 75-80% of their initial weight, they were habituated to handling. The liquid reward for handling and training consisted of 15.5 g powdered baby soymilk (SMA Nutrition) and 2g of sugar per 100 ml of water. Task training began when

animals stopped showing signs of distress when being handled (generally after 1-4 handling sessions).

Systematic literature review

The studies included in Figures 2f and 3e were identified by a systematic literature search of the Pubmed and Medline databases, conducted in August 2015. The search strategy was agreed upon before the search, and was based on a filter as described by Hooijmans et al. (2010)[2], adapted to mice and including several additional keywords such as visual stimulus, visual discrimination, visual detection and derivatives of those. The initial search resulted in 75 articles. Since we were keen to highlight recent developments in behavioural paradigms for mice, we included studies from December 2011 until December 2016. We also excluded studies conducted in mouse models of disease (e.g. reduced vision or learning impairments) if they failed to report a full set of results for a control group of wild-type animals. For the studies included in the analysis of training speed (Fig. 2f), an additional requirement was that they needed to specifically report the number of trials and/or training sessions necessary to reach criterion performance (rather than referring to training times of 'several weeks' or the like). This strategy resulted in a final set of 18 studies included in Figure 2f, and 31 studies in Figure 3e. The studies were:

1 Barkus, C. *et al.* Do GluA1 knockout mice exhibit behavioral abnormalities relevant to the negative or cognitive symptoms of schizophrenia and schizoaffective disorder? *Neuropharmacology* **62**, 1263-1272, doi:10.1016/j.neuropharm.2011.06.005 (2012).

2 Bennett, C., Arroyo, S. & Hestrin, S. Subthreshold mechanisms underlying state-dependent modulation of visual responses. *Neuron* **80**, 350-357, doi:10.1016/j.neuron.2013.08.007 (2013).

3 Bubser, M. *et al.* Selective activation of M4 muscarinic acetylcholine receptors reverses MK-801-induced behavioral impairments and enhances associative learning in rodents. *ACS Chem Neurosci* **5**, 920-942, doi:10.1021/cn500128b (2014).

4 Carr, G. V., Jenkins, K. A., Weinberger, D. R. & Papaleo, F. Loss of dysbindin-1 in mice impairs reward-based operant learning by increasing impulsive and compulsive behavior. *Behav Brain Res* **241**, 173-184, doi:10.1016/j.bbr.2012.12.021 (2013).

5 Cole, R. D., Poole, R. L., Guzman, D. M., Gould, T. J. & Parikh, V. Contributions of beta2 subunit-containing nAChRs to chronic nicotine-induced alterations in cognitive

flexibility in mice. *Psychopharmacology (Berl)* **232**, 1207-1217, doi:10.1007/s00213-014-3754-4 (2015).
6 Dickson, P. E., Calton, M. A. & Mittleman, G. Performance of C57BL/6J and DBA/2J mice on a touchscreen-based attentional set-shifting task. *Behav Brain Res* **261**, 158-170, doi:10.1016/j.bbr.2013.12.015 (2014).
7 Dickson, P. E. *et al.* Effects of stimulus salience on touchscreen serial reversal learning in a mouse model of fragile X syndrome. *Behav Brain Res* **252**, 126-135, doi:10.1016/j.bbr.2013.05.060 (2013).
8 Gabel, L. A. *et al.* Mutation of the dyslexia-associated gene Dcdc2 impairs LTM and visuo-spatial performance in mice. *Genes Brain Behav* **10**, 868-875, doi:10.1111/j.1601-183X.2011.00727.x (2011).
9 Glickfeld, L. L., Histed, M. H. & Maunsell, J. H. Mouse primary visual cortex is used to detect both orientation and contrast changes. *J Neurosci* **33**, 19416-19422, doi:10.1523/JNEUROSCI.3560-13.2013 (2013).
10 Graybeal, C. *et al.* Strains and stressors: an analysis of touchscreen learning in genetically diverse mouse strains. *PLoS One* **9**, e87745, doi:10.1371/journal.pone.0087745 (2014).
11 Histed, M. H., Carvalho, L. A. & Maunsell, J. H. Psychophysical measurement of contrast sensitivity in the behaving mouse. *J Neurophysiol* **107**, 758-765, doi:10.1152/jn.00609.2011 (2012).
12 Humby, T., Eddy, J. B., Good, M. A., Reichelt, A. C. & Wilkinson, L. S. A novel translational assay of response inhibition and impulsivity: effects of prefrontal cortex lesions, drugs used in ADHD, and serotonin 2C receptor antagonism. *Neuropsychopharmacology* **38**, 2150-2159, doi:10.1038/npp.2013.112 (2013).
13 Kahn, J. B. *et al.* Medial prefrontal lesions in mice impair sustained attention but spare maintenance of information in working memory. *Learn Mem* **19**, 513-517, doi:10.1101/lm.026302.112 (2012).
14 Kato, S. *et al.* Selective neural pathway targeting reveals key roles of thalamostriatal projection in the control of visual discrimination. *J Neurosci* **31**, 17169-17179, doi:10.1523/JNEUROSCI.4005-11.2011 (2011).
15 Kim, C. H., Heath, C. J., Kent, B. A., Bussey, T. J. & Saksida, L. M. The role of the dorsal hippocampus in two versions of the touchscreen automated paired associates learning (PAL) task for mice. *Psychopharmacology (Berl)* **232**, 3899-3910, doi:10.1007/s00213-015-3949-3 (2015).
16 Kolisnyk, B. *et al.* Forebrain deletion of the vesicular acetylcholine transporter results in deficits in executive function, metabolic, and RNA splicing abnormalities in the prefrontal cortex. *J Neurosci* **33**, 14908-14920, doi:10.1523/JNEUROSCI.1933-13.2013 (2013).
17 Lehmann, K., Schmidt, K. F. & Lowel, S. Vision and visual plasticity in ageing mice. *Restor Neurol Neurosci* **30**, 161-178, doi:10.3233/RNN-2012-110192 (2012).
18 Lyon, L. *et al.* Fractionation of spatial memory in GRM2/3 (mGlu2/mGlu3) double knockout mice reveals a role for group II metabotropic glutamate receptors at the interface between arousal and cognition. *Neuropsychopharmacology* **36**, 2616-2628, doi:10.1038/npp.2011.145 (2011).

19 Makowiecki, K., Hammond, G. & Rodger, J. Different levels of food restriction reveal genotype-specific differences in learning a visual discrimination task. *PLoS One* **7**, e48703, doi:10.1371/journal.pone.0048703 (2012).
20 Mar, A. C. *et al.* The touchscreen operant platform for assessing executive function in rats and mice. *Nat Protoc* **8**, 1985-2005, doi:10.1038/nprot.2013.123 (2013).
21 Marquardt, K., Saha, M., Mishina, M., Young, J. W. & Brigman, J. L. Loss of GluN2A-containing NMDA receptors impairs extra-dimensional set-shifting. *Genes Brain Behav* **13**, 611-617, doi:10.1111/gbb.12156 (2014).
22 Okada, K. *et al.* Enhanced flexibility of place discrimination learning by targeting striatal cholinergic interneurons. *Nat Commun* **5**, 3778, doi:10.1038/ncomms4778 (2014).
23 Ortega, L. A., Tracy, B. A., Gould, T. J. & Parikh, V. Effects of chronic low- and high-dose nicotine on cognitive flexibility in C57BL/6J mice. *Behav Brain Res* **238**, 134-145, doi:10.1016/j.bbr.2012.10.032 (2013).
24 Pinto, L. *et al.* Fast modulation of visual perception by basal forebrain cholinergic neurons. *Nat Neurosci* **16**, 1857-1863, doi:10.1038/nn.3552 (2013).
25 Poort, J. *et al.* Learning Enhances Sensory and Multiple Non-sensory Representations in Primary Visual Cortex. *Neuron* **86**, 1478-1490, doi:10.1016/j.neuron.2015.05.037 (2015).
26 Rutz, H. L. & Rothblat, L. A. Intact and impaired executive abilities in the BTBR mouse model of autism. *Behav Brain Res* **234**, 33-37, doi:10.1016/j.bbr.2012.05.048 (2012).
27 Silverman, J. L., Gastrell, P. T., Karras, M. N., Solomon, M. & Crawley, J. N. Cognitive abilities on transitive inference using a novel touchscreen technology for mice. *Cereb Cortex* **25**, 1133-1142, doi:10.1093/cercor/bht293 (2015).
28 Sykes, M., Makowiecki, K. & Rodger, J. Long term delivery of pulsed magnetic fields does not alter visual discrimination learning or dendritic spine density in the mouse CA1 pyramidal or dentate gyrus neurons. *F1000Res* **2**, 180, doi:10.12688/f1000research.2-180.v2 (2013).
29 Trevino, M. Stimulus similarity determines the prevalence of behavioral laterality in a visual discrimination task for mice. *Sci Rep* **4**, 7569, doi:10.1038/srep07569 (2014).
30 Trevino, M. *et al.* Controlled variations in stimulus similarity during learning determine visual discrimination capacity in freely moving mice. *Sci Rep* **3**, 1048, doi:10.1038/srep01048 (2013).
31 Zhang, S. *et al.* Selective attention. Long-range and local circuits for top-down modulation of visual cortex processing. *Science* **345**, 660-665, doi:10.1126/science.1254126 (2014).

Additional performance metrics

In addition to the seven primary metrics discussed in the main manuscript, we computed four further metrics, which were not shown in the main text for the sake of brevity, but may also be useful in different experimental contexts:

1) Distractor distance

The distractor distance was computed in the same way as the target distance, but relative to the lateral position of the distractor. Since there were only one target and one distractor to choose from in this paradigm, target distance and distractor distance were largely redundant. However, in configurations with multiple and/or non-simultaneously appearing targets and/or distractors, this measure would be useful.

2) Time-to-target

The time-to-target was simply the time the animal took from the moment the target shifted until reaching the target. Since animals tended to slow down just before the target (e.g. in order to start licking), we actually defined the moment of 'reaching the target' as the first time animals came within 10 cm of any target position. Given that the time-to-target could be largely predicted based on the reaction time, running speed and path efficiency, it was redundant in the current context, however it can be a good 'summary measure' of the efficiency with which animals reach different targets.

3) Lick timing

As a second measure of reward anticipation, we quantified the timing of licks relative to the trial end (i.e. the reward time in case of correct trials). For each trial, we took into account 2.5 seconds before and after the trial offset. An exception was made if the current trial was shorter than 2.5 seconds, in which case we quantified lick times beginning with the trial onset + 0.5 seconds; and if the following trial was shorter than 2.5 seconds, we quantified lick times up to the following trial offset – 0.5 seconds. This time buffer was introduced because at the beginning of the trial and at

the end of the next trial you would expect the animal to be licking in response to the previous/following reward. The average lick time per trial was then computed as the mean of the included lick times relative to the trial offset. To compute a mean lick time across trials, lick times were first pooled across trials and then averaged.

While lick timing would seem to be the more intuitive measure of reward anticipation compared to 'lick position', in this manuscript we used lick position rather than lick timing as the main indicator of licking behaviour. The reason is that animals tended to begin licking at specific locations, mostly right in front of the target; and as a result, lick times varied mainly depending on the animal's running speed across trials, which determined the time delay between a largely consistent lick location and the trial end. Thus, in the present configuration lick timing tended to underestimate the clustering of licking behaviour around the target location.

4) Side bias

Like the local PR score, we computed estimates of side bias as a running average across small trial sets in order to test whether animals chose left/right targets preferentially throughout the course of training. Side bias could be estimated based on each performance metric.

Since side biases could change, for instance, over the course of a session, it was important to compare trials from approximately the same time point. We therefore computed the side bias using a sliding window analysis, creating separate running averages of performance for trials featuring left targets and right targets. The sliding analysis window labelled by index *i* took into account 25 consecutive trials featuring the same target position, and was moved in steps of one trial. Based on the two resulting M vectors containing the averaged performance for left and right targets

over time, we then computed the side bias for each vector entry in the following way:

$$SB_i = \frac{M_{L,i} - M_{R,i}}{|M_{L,i}| + |M_{R,i}|} \quad (16)$$

where SB_i is the side bias for vector entry i, $M_{L,i}$ is the running average of performance measure M for vector entry i based on left-target trials, and $M_{R,i}$ is the corresponding measure for right-target trials. The resulting side bias could take values between -1 and 1, with -1 indicating a larger performance measure for right-hand trials, 1 indicating a larger performance measure for left-hand trials, and 0 indicating balance between left and right trials. Note that since trials were pseudo-randomized (meaning that the proportion of different trial types evened out in chunks of 10 trials), a running average pooling 25 left-target trials and a running average pooling 25 right-target trials will refer to an almost identical time window within the session.

Additional statistics for supplementary figures

Testing samples using a common test (z) distribution across ΔOri (Fig. S5b)

Assessing the statistical significance of the changes in CL index shown in Fig. S5b posed several challenges: The CL index changes for trials with similar ΔOri were not independent from each other: Similar ΔOri showed similar changes in CL index and more dissimilar ΔOri often showed consistently opposite changes. Using a t-test/ANOVA treating all ΔOri as independent comparisons would therefore not do justice to this constellation. Yet changes for individual ΔOri clearly had to be tested individually, since pooling them would wash out performance changes. Moreover, with four and five animals, respectively, the sample size was extremely low particularly for cued changes in CL index, meaning that e.g. t-tests with

correction for multiple comparisons would have very low statistical power. We therefore decided to use a z-test to assess the performance change for every ΔOri in relation to a common test distribution. The test distribution was defined to have a mean of zero (i.e. 'no performance change'). The error variance of the test distribution was derived from the error variances across all ΔOri as follows: For each ΔOri, we subtracted the mean from all individual measurements. We then pooled the mean-subtracted data across all ΔOri, and computed their standard deviation. The measurements for each ΔOri were then tested against the resulting z distribution.

Supplementary Note 1: Addressing confounds in attention tasks for mice

Based on the list of potential confounds in Figure 1 and Table 1, we here discuss in more detail how and to what extent such confounds can be addressed by different attention tasks for mice. To make a representative but confined comparison, we compare the VEF task presented here with three popular paradigms: The 5-choice-serial reaction time task (5CSRTT), the continuous performance task (CPT) and an attentional set shifting task (SST). Since there are different implementations of these tasks, we assume that the 5CSRTT and the SST are implemented in an operant conditioning (OC) box, whereby an animal has to touch the correct target on a touchscreen (see e.g.[3-9]). For balance, we refer to an implementation of the CPT that is more controlled – with stimuli being presented on a screen, and animals being largely immobile, responding with a lick/nose poke response [10,11].

1) Stimulus presentation

In an ideal paradigm, both the timing of stimuli relative to the animal's actions and the position of the stimulus relative to the animal's viewpoint would be completely controlled. In both respects, tasks implemented in an OC box offer poor control of stimulus presentation: Neither the animal's position, direction of gaze nor occupation (e.g. grooming, running, eating) at the point of stimulus onset are controlled, and could only be extracted for analysis by manually scoring the corresponding video footage. In a head-fixed CPT task, such confounds (except for grooming and eye movements – see below) are largely eliminated. Similarly, in the VEF task, the head-fixed animal is 'reset' to the exact same position in the virtual environment at each trial onset, meaning that the stimulus appears at exactly the same position relative to the animal's position and visual field each time. The only source of variability are eye movements, which are reasonably rare in mice in this context [12], and can be measured and accounted for if necessary [12].

2) Visual acuity

Neither 5CSRTT, CPT nor SST contain explicit measures of visual acuity. Yet it stands to reason that animals with low visual acuity will have more difficulty detecting a stimulus appearance (5CSRTT), stimulus change (CPT) or identifying a stimulus difference (SST) than animals with high visual acuity. The only way in which this issue is addressed to some extent is in task implementations that show large, simple stimuli. However, not all task implementations use simple stimuli, and even in such cases, this does not solve the issue for low-vision animals. In general, low visual acuity is therefore likely to result in lower attention scores for all three comparison tasks. In the VEF task, visual acuity is explicitly measured, and has been demonstrated to be independent of our metrics of sustained *and* cued attention (see Supp. Fig. S7). This is most likely due to the fact that both sATT score and cATT score do not rely on absolute performance levels, eliminating the influence of visual acuity on test outcomes: The sATT score quantifies the amount of time spent in High-Alert states, irrespective of such High-Alert states lead to perfect or less-than-perfect performance in different animals. The cATT score measures the performance difference between cued and non-cued trials, rather than absolute performance.

3) Overtraining

5CSRTT, CPT and SST all require at least several weeks of task training – ample time to substantially alter cortical circuit function through long-term plasticity (see e.g. [13]). The main reason that such extensive training is needed is not the task in itself but essentially unrelated challenges: In the case of the OC box, the entire operation of the apparatus, as well as the association between touching an object based solely on visual cues, and then collecting food reward from an entirely different object. In the case of head fixation, operation of the response unit (e.g. a lick port), and most importantly, getting used to being restrained for prolonged periods of time without the typical stress response. The VEF task

largely removes such non-essential obstacles by using an immersive spatial environment, a response scheme designed to approximate foraging (reward is available immediately upon approaching the correct object), and minimizing the movement restraint for animals. This approach cuts training times to 3-5 days, largely eliminating the issue of overtraining.

4) Rule learning and memory capacity

In terms of the cognitive processes involved, performing an attention-based task is a very different challenge from learning that task. Task learning requires memory, pattern recognition (e.g. 'food pellets appear after pressing button X'), outcome valuation (positive affect towards reward, negative affect towards punishment) and a host of other processes that can all be conceptually dissociated from attention itself. Any animal that fails at one or more of these unrelated processes will therefore by definition fail to achieve high performance in the attention task it is supposed to acquire. The more complex a task, the larger the confound.

While all three comparison tasks are heavy on non-task-related complexity (e.g. in terms of response schemes, see point 3), the 5CSRTT and CPT at least feature reasonably simple task rules ('Move towards the location where a stimulus appears.' and 'Respond if you notice a stimulus change.'). In contrast, the SST requires more complex rule learning and memory – memorizing two different task rules, as well as the context in which they apply. This implies that SSTs are unlikely to yield performance metrics that solely reflect attention, as evidenced e.g. by the fact that mice are able to perform set shifting across sensory modalities with much greater ease compared to set shifting within the visual domain[5,12,14-16]. In theory, these different task implementations should measure the same attentional capacity, yet performance is clearly dominated by other factors.

The VEF task addresses the confound of rule learning and memory capacity in three ways: It minimizes the complexity of the task rule itself ('Approach rewarded object, ignore all else.');

it removes non-task-related complexity (see point 3 for details), and it measures the moment of rule acquisition explicitly and independently of rule performance [1]. Trials that occurred before the point of rule acquisition can therefore simply be excluded post-hoc from the computation of attention metrics.

5) Cognitive Flexibility

Cognitive flexibility refers to the ability to dynamically adjust behavioural responses to different contexts. As such, it draws on cognitive factors like recognizing different contexts as well as motivational factors like an absence of obsessive-compulsive traits. While 5CSRTT, CPT and VEF task all place low demands on cognitive flexibility by featuring only one task rule, the central requirement of the SST is the flexible transition from one response scheme to another – a classical gauge of cognitive flexibility. In fact, the same SSTs are independently used to measure attention and cognitive flexibility by different studies [15,17-19]. This makes SSTs considerably more affected by confounds of cognitive flexibility than the other three tasks.

6) Speed-accuracy bias

Trade-offs between speedy and accurate behavioural responses are ubiquitous across species [20-24]. As such, an attention task that does not take speed-accuracy trade-offs into account will run the danger of classifying low-performing animals in the same way as animals that are being attentive, but prioritize respond speed over accuracy. This is indeed the case for the SST: Animals that prioritize response speed are likely to make more errors during set switches, leading to lowered attention scores. In the case of 5CSRTT and CPT, this confound also poses a problem, but in the opposite direction: Since in these tasks inattention is defined by the percentage of omitted responses, animals that prioritize fast over accurate responses will on average attain better scores, since they are less likely to

omit trials they are uncertain of. The VEF task does not feature a classical trade-off between reaction time and hit rate at all because the moment of the first stimulus response (reaction time), and the moment of the final target choice (hit rate) are decoupled (see Supp. Fig. S6). The speed-accuracy trade-off that it does invoke – the trade-off between reaction times and path surplus – can be explicitly measured, and can actually be used to directly classify animals as high, low, impulsive and thorough performers, making it easy to address this confound post-hoc (Supp. Fig. S6).

7) Motor difficulty (e.g. training/impairment/fatigue)

Any behavioural task by definition relies on an animal's motor output to infer underlying perceptual or cognitive processes. This poses the question how much response variability is due to the perceptual/cognitive process in question, and how much is produced by factors that arise purely from the difficulty of the required motor output. To some extent this is clearly a concern for all tasks. However, tasks requiring simple, reflexive, low-strain responses like licking (e.g. the CPT task) do fare better in this respect: Animals clearly don't need much training or stamina in order to lick a reward spout. The challenge in such cases lies conversely in the fact that the motor response is so easy that animals struggle to inhibit their responses (see point 8). 5CSRTT and SST, in their implementation in OC boxes, are more likely to be contaminated by motor difficulty because they require a good amount of running. This means that animals who are fatigued or otherwise impaired in their locomotion will most likely incur a larger number of omitted responses. In the 5CSRTT, this directly leads to a lowered attention score, and motor impairments can generally not be analysed or taken into account post-hoc. In the SST, motor difficulties would most likely simply reduce the number of completed trials. The VEF task also requires running, and as such is subject to similar issues. The fact that running takes place on a treadmill does not seem to increase motor difficulty in any significant way: Animals learn to run on the treadmill within a few

minutes of the first session, and learn to steer within 15-30 minutes after lateral targets are introduced. They also keep running even after the session has been terminated, indicating that movement on the treadmill is intuitive, easy to learn, and does generally not lead to fatigue. In contrast to tasks that feature running in an OC box, the VEF task offers the advantage that fatigue or motor impairments can be registered explicitly – most simply by tracking running speed, but if necessary also by analysing running paths in more detail in order to detect whether changes in running direction are executed neatly.

8) Response inhibition

While tasks with a simple response modality (e.g. licking) minimize confounds related to motor difficulty (see point 7), they face a different obstacle: The need for animals to inhibit random responses. In the absence of displacement activity like running or grooming, inhibiting behavioural responses is such a challenge for mice that large amounts of training and punishment are required in order to learn it [e.g. 11,25-27]. This implies that even after extensive training, some task responses may still simply be a product of failed response inhibition. In this respect, simple responses like licking incur a double penalty: They are so metabolically and cognitively cheap that response inhibition becomes very difficult for animals; and the binary nature of the response makes it impossible to distinguish between deliberate and random responses in post-hoc analysis. Tasks that require a more protracted response, like running in an OC box, will most likely invite less random activity, but there is again no way of explicitly identifying such random responses. The presence of false-positive responses in the 5CSRTT would suggest that at least some of them are due to failed response inhibition. The SST faces fewer problems with this confound because it relies not only on a somewhat demanding motor response (running), but also on a forced-choice paradigm. This means that animals generally do not need to inhibit responses, they just need to choose between one and another. The VEF task tackles the confound of response

inhibition from several angles: First, animals are constantly running on the treadmill, so that displacement activity is available in the absence of a task response. Second, responses in the form of steering towards one side are prolonged and quite costly. As a consequence, in the absence of a visible target stimulus, animals showed a false positive rate of zero, suggesting that targets are only approached as a deliberate choice, and not due to failed response inhibition. Finally, if one wanted to nevertheless control for non-target-related responses, the analysis of anticipatory licking can help to identify responses for which the animal did not expect to be rewarded (see point 11).

9) Motivation

While attention and motivation certainly are interdependent processes (no attention without motivation), they should nevertheless be distinguished conceptually. In particular, a lack of motivation should ideally not be mistaken for low attentional capacity. This problem is not addressed at all in the 5CSRTT and CPT, where low motivation (e.g. satiation from food pellets) would directly lead to more omitted responses and therefore lower attention scores. The influence of such fluctuations in motivation can be observed in action by tracking the rate of false positives over the course of a session: In many paradigms, false positives drop off throughout a session, indicating that animals become progressively more comfortable with missing rewards – likely due to satiation. The impact of low motivation on performance in the SST is not quite as obvious, but it is also not accounted for explicitly. In the VEF task, motivation is tracked via running speed and licking frequency – when animals slow down, begin to take breaks in running, or lick less frequently once reward is delivered, the session is terminated. By tracking these indicators of motivation, we have found that relying on food rather than water deprivation yields a 'flatter' motivation curve throughout a session: Food-deprived animals are less over-eager at the start of a session, and stay more motivated over

time, than water-deprived animals. In this way, the VEF task ensures a largely constant level of good motivation throughout a session.

10) Response omissions

When analysing attentive behaviour, it is important to distinguish between omitted/missed responses, and active trial rejection. In both the 5CSRTT and the CPT this is not a given: If an animal does not respond to the appearance/change of a stimulus, there is no way to determine whether it has actively rejected the option that the stimulus appeared/changed (e.g. due to low visual acuity, see point 2), or whether it simply missed the response – which would be a sign of inattention. As a forced-choice paradigm, the SST is less affected by omitted responses, but simply ignores them. In the VEF task, although animals are asked to choose between two targets, they also have the option to run between targets, effectively giving a no-choice response. However, no-choice trials will be followed by a time-out corridor, and repeated until the correct target choice is made. As a consequence, animals are trained not to omit responses randomly, given the metabolic cost of completing the time-out corridor and subsequently repeating the trial. As such, trials in which animals do not hit either target can be largely regarded as the product of actual choice uncertainty rather than missing the trial (see point 11). This interpretation is supported by the fact that the number of no-choice trials increases with stimulus difficulty, indicating that animals do not randomly omit target responses.

11) Uncertain/random choices and accidental responses

This point refers to a range of task responses that are not based on a deliberate target choice. For instance, an animal may choose a response randomly due to stimulus uncertainty or lacking motivation, or may accidentally enact an unintended response (e.g. licking the wrong lick spout). Similarly to omissions, random/uncertain or accidental

responses should be distinguishable from deliberate but erroneous choices. This confound is most critical for the CPT: Since licking is such a quick and simple action, accidental as well as random licking can easily occur, and would not be distinguishable from a deliberate response (see point 8). Both 5CSRTT and SST profit from the fact that the required motor response (running towards a target) is somewhat more prolonged, and therefore makes accidental responses less likely. However, this does not preclude random choices, as shown by the ubiquity of trials with premature responses and wildly missed targets (e.g. choosing target position 1 when target position 5 displayed a stimulus) in the 5CSRTT. For the SST, random choices are an even greater issue, since animals are likely to be highly uncertain of their target choices after a set shift. In both tasks, such random choices are difficult to identify post-hoc. The VEF task tackles the issue of random and accidental responses in two ways: First, by requiring a challenging and prolonged change in running direction, accidental target choices are eliminated. Random target choices are also vastly reduced - by the metabolic cost of the response itself, by the cost of the time-out corridor and the trial repetition that follows an incorrect choice, and by the option to give a no-choice response in cases of uncertainty (see point 10). Finally, the remaining uncertain or random choices can be pinpointed post-hoc by analysing the degree of anticipatory licking: When animals are not confident of their target choice, they show a marked reduction of anticipatory licking before they reach the target.

Supplementary References

1 Havenith, M. N. *et al.* Rapid training and single-trial metrics: A visual task to measure rule acquisition and reversal in head-fixed mice. Submiited as a linked manuscript to *Nature Scientific Reports*.

2 Hooijmans, C. R., Leenaars, M. & Ritskes-Hoitinga, M. A gold standard publication checklist to improve the quality of animal studies, to fully integrate the Three Rs, and to make systematic reviews more feasible. *Altern Lab Anim* **38**, 167-182 (2010).

3 Young, J. W., Light, G. A., Marston, H. M., Sharp, R. & Geyer, M. A. The 5-choice continuous performance test: evidence for a translational test of vigilance for mice. *PLoS One* **4**, e4227, doi:10.1371/journal.pone.0004227 (2009).

4 Kim, H., Ahrlund-Richter, S., Wang, X., Deisseroth, K. & Carlen, M. Prefrontal Parvalbumin Neurons in Control of Attention. *Cell* **164**, 208-218, doi:10.1016/j.cell.2015.11.038 (2016).

5 Brigman, J. L., Bussey, T. J., Saksida, L. M. & Rothblat, L. A. Discrimination of multidimensional visual stimuli by mice: intra- and extradimensional shifts. *Behav Neurosci* **119**, 839-842, doi:10.1037/0735-7044.119.3.839 (2005).

6 Bussey, T. J. *et al.* The touchscreen cognitive testing method for rodents: how to get the best out of your rat. *Learn Mem* **15**, 516-523, doi:10.1101/lm.987808 (2008).

7 Kim, C. H. *et al.* The continuous performance test (rCPT) for mice: a novel operant touchscreen test of attentional function. *Psychopharmacology (Berl)* **232**, 3947-3966, doi:10.1007/s00213-015-4081-0 (2015).

8 Hvoslef-Eide, M., Nilsson, S. R., Saksida, L. M. & Bussey, T. J. Cognitive Translation Using the Rodent Touchscreen Testing Approach. *Curr Top Behav Neurosci* **28**, 423-447, doi:10.1007/7854_2015_5007 (2016).

9 Guillem, K. *et al.* Nicotinic acetylcholine receptor beta2 subunits in the medial prefrontal cortex control attention. *Science* **333**, 888-891, doi:10.1126/science.1207079 (2011).

10 Gritton, H. J. *et al.* Cortical cholinergic signaling controls the detection of cues. *Proc Natl Acad Sci U S A* **113**, E1089-1097, doi:10.1073/pnas.1516134113 (2016).

11 Zhang, S. *et al.* Selective attention. Long-range and local circuits for top-down modulation of visual cortex processing. *Science* **345**, 660-665, doi:10.1126/science.1254126 (2014).

12 Poort, J. *et al.* Learning Enhances Sensory and Multiple Non-sensory Representations in Primary Visual Cortex. *Neuron* **86**, 1478-1490, doi:10.1016/j.neuron.2015.05.037 (2015).

13 Kawai, R. *et al.* Motor cortex is required for learning but not for executing a motor skill. *Neuron* **86**, 800-812, doi:10.1016/j.neuron.2015.03.024 (2015).

14 Wimmer, R. D. *et al.* Thalamic control of sensory selection in divided attention. *Nature* **526**, 705-709, doi:10.1038/nature15398 (2015).

15 Bissonette, G. B. & Powell, E. M. Reversal learning and attentional set-shifting in mice. *Neuropharmacology* **62**, 1168-1174, doi:10.1016/j.neuropharm.2011.03.011 (2012).

16 Garner, J. P., Thogerson, C. M., Wurbel, H., Murray, J. D. & Mench, J. A. Animal neuropsychology: validation of the Intra-Dimensional Extra-Dimensional set shifting task for mice. *Behav Brain Res* **173**, 53-61, doi:10.1016/j.bbr.2006.06.002 (2006).

17 Birrell, J. M. & Brown, V. J. Medial frontal cortex mediates perceptual attentional set shifting in the rat. *J Neurosci* **20**, 4320-4324 (2000).
18 Brown, V. J. & Tait, D. S. Attentional Set-Shifting Across Species. *Curr Top Behav Neurosci* **28**, 363-395, doi:10.1007/7854_2015_5002 (2016).
19 Colacicco, G., Welzl, H., Lipp, H. P. & Wurbel, H. Attentional set-shifting in mice: modification of a rat paradigm, and evidence for strain-dependent variation. *Behav Brain Res* **132**, 95-102 (2002).
20 Lyons, J. J. & Briggs, G. E. Speed-accuracy trade-off with different types of stimuli. *J Exp Psychol* **91**, 115-119 (1971).
21 Chittka, L., Skorupski, P. & Raine, N. E. Speed-accuracy tradeoffs in animal decision making. *Trends Ecol Evol* **24**, 400-407, doi:10.1016/j.tree.2009.02.010 (2009).
22 Heitz, R. P. The speed-accuracy tradeoff: history, physiology, methodology, and behavior. *Front Neurosci* **8**, 150, doi:10.3389/fnins.2014.00150 (2014).
23 Drugowitsch, J., DeAngelis, G. C., Angelaki, D. E. & Pouget, A. Tuning the speed-accuracy trade-off to maximize reward rate in multisensory decision-making. *Elife* **4**, e06678, doi:10.7554/eLife.06678 (2015).
24 Spieser, L., Servant, M., Hasbroucq, T. & Burle, B. Beyond decision! Motor contribution to speed-accuracy trade-off in decision-making. *Psychon Bull Rev*, doi:10.3758/s13423-016-1172-9 (2016).
25 Pinto, L. *et al.* Fast modulation of visual perception by basal forebrain cholinergic neurons. *Nat Neurosci* **16**, 1857-1863, doi:10.1038/nn.3552 (2013).
26 Andermann, M. L., Kerlin, A. M. & Reid, R. C. Chronic cellular imaging of mouse visual cortex during operant behavior and passive viewing. *Front Cell Neurosci* **4**, 3, doi:10.3389/fncel.2010.00003 (2010).
27 Histed, M. H., Carvalho, L. A. & Maunsell, J. H. Psychophysical measurement of contrast sensitivity in the behaving mouse. *J Neurophysiol* **107**, 758-765, doi:10.1152/jn.00609.2011 (2012).
28 Carandini, M. & Churchland, A. K. Probing perceptual decisions in rodents. *Nat Neurosci* **16**, 824-831, doi:10.1038/nn.3410 (2013).

Figure S1: Primary performance metrics for one example session

a) Seven primary single-trial performance metrics (see Methods) derived from the running trajectories of the training session shown in Figs. 3a-c. Circles: Single-trial metrics. Black line: 15-trial running average. Red lines: Time points at which more difficult **Δ**Ori between target and distractor were introduced (**Δ**Ori introduced at a given time point are indicated above left upper panel). Right-hand side insets: Distribution of each performance measure across the training session. Some measures (e.g. path surplus and lick position) yielded wide, largely normal distributions across the session. Other measures, for instance target distance and PR score, were distributed less evenly. PR scores in particular showed rhythmic variations over time, resulting in an overall bimodal distribution (for further analysis of this phenomenon, see Fig. 5 and Supp. Figs. S3-S4).

b) Running trajectories of one animal completing the task with the virtual-environment projection switched off. While paths can deviate slightly from the center, no path shows the sudden change in running direction that would be necessary to capture a target. As a result, the animal's (false positive) hit rate under these circumstances is zero.

Figure S1

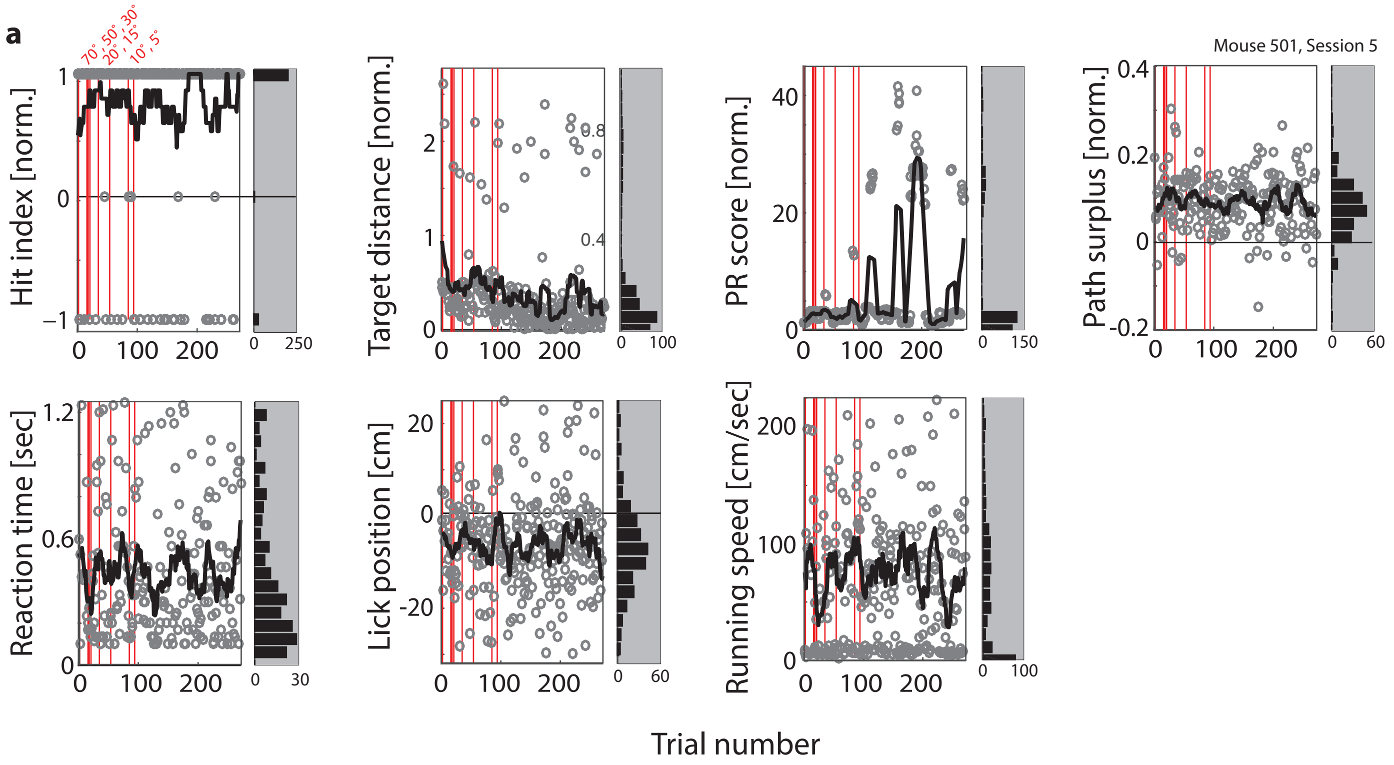
a
Mouse 501, Session 5
70°, 50°, 30°
20°, 15°
10°, 5°
Hit index [norm.]
Target distance [norm.]
PR score [norm.]
Path surplus [norm.]
Reaction time [sec]
Lick position [cm]
Running speed [cm/sec]
Trial number

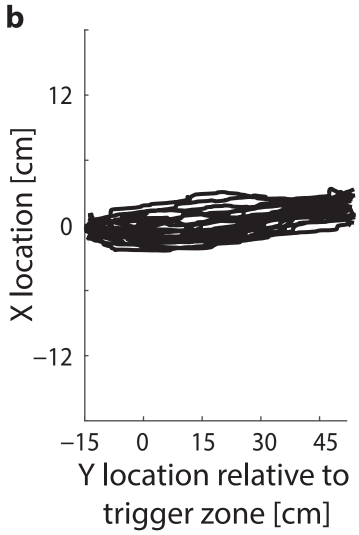
b
X location [cm]
Y location relative to trigger zone [cm]

Figure S2: Mice perform consistently close to the limit of their ability

a) Left column: Distributions of five representative primary metrics of performance for Mouse 204. Running speed and lick position are not shown because they represent largely stimulus-independent metrics of overall motivation, rather than processing capacity or task performance. Trials from training stage 6 were included. Red: Performance for the two easiest stimulus conditions (ΔOri ≥ 70°). Black: Performance for the remaining stimulus conditions (ΔOri < 70°). Dashed lines: Cut-off criteria for significant orientation discrimination ($\alpha = 0.05$) for hit index, target distance and PR score. Right column: Same for Mouse 601. In easy stimulus conditions, both animals showed sharp distributions close to optimal performance across a number of metrics: The hit index was consistently close to 1, and never fell below the threshold for significant stimulus discrimination. Even more strikingly, the target distance remained firmly below 1 for virtually all easy trials, meaning that even in error trials, animals were steering in the overall direction of the correct target and only missed it by a small distance. Path surplus also showed sharp peaks very close to optimal performance, and the vast majority of PR scores far exceeded the discrimination criterion. Reaction times for easy trials also showed a clear peak around optimal speed. Note that Mouse 204 seems to respond generally somewhat slower than Mouse 601, but in both cases, easy trials produced a dominant peak very close to the fastest time each animal was able to reach. Both peaks centre around 200-300 ms, making both distributions very similar to typical response times for button press or lick responses (see e.g.[28]).

b) Same as a, but for trials pooled across all 12 animals, showing the same effects as discussed for the example animals in A. Note that the effect for reaction times is somewhat 'blurred' compared to the individual distributions in A, most likely due to the fact that the distributions are pooled across different animals with different maximal reaction speeds.

Figure S2

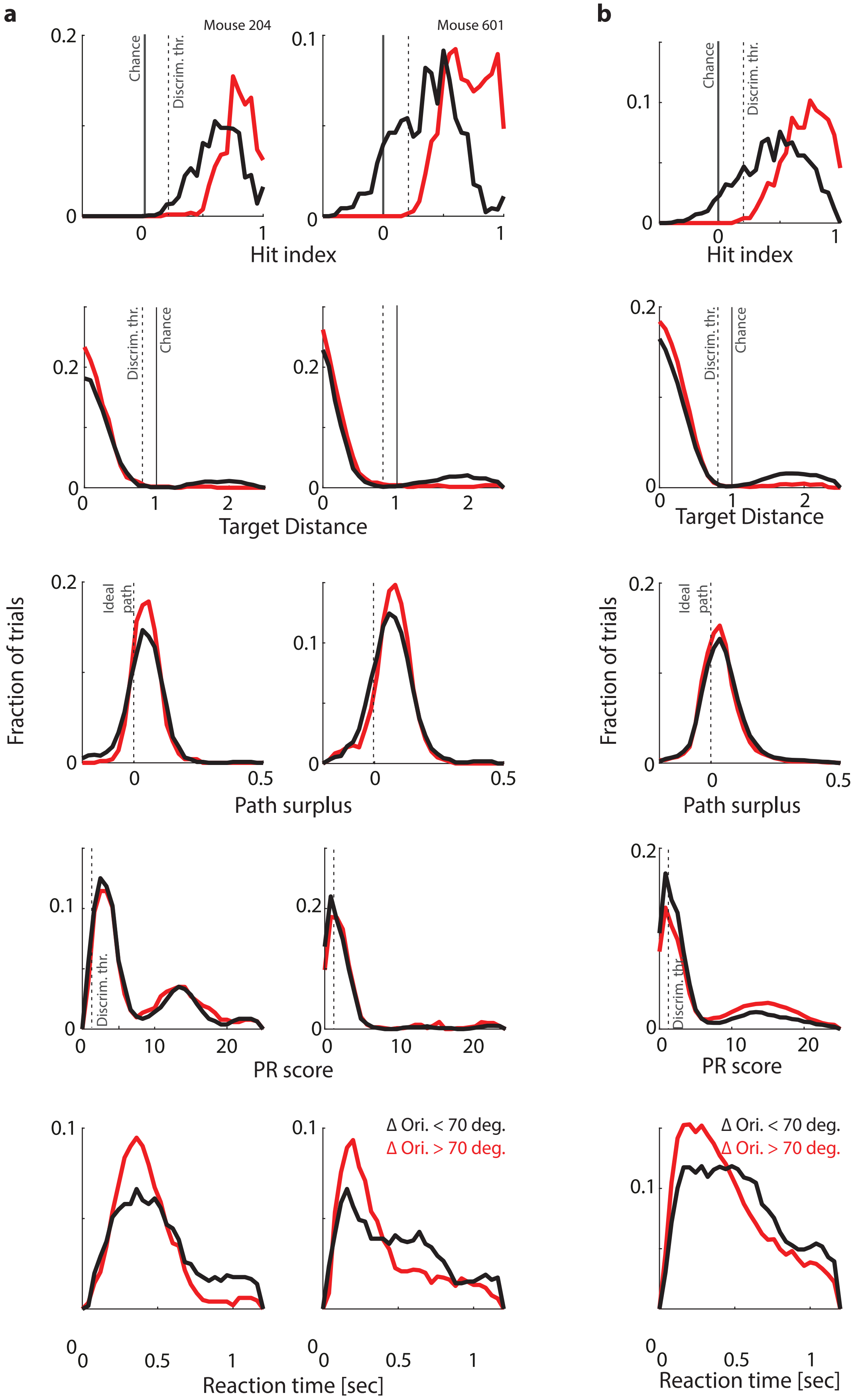
a
Mouse 204
Mouse 601
b
Chance
Discrim. thr.
Hit index
Target Distance
Ideal path
Fraction of trials
Path surplus
PR score
Δ Ori. < 70 deg.
Δ Ori. > 70 deg.
Reaction time [sec]

Figure S3: The PR score mirrors shifts between high and low alertness

a) Distribution of bimodality coefficients across animals. Dots: Bimodality coefficient per animal, computed for the distribution of local PR scores across trials in the final training stage (see Fig. 5a). Red lines: Mean across animals ± St.Dev. Dashed line: Critical threshold (0.55) for accepting distributions as bimodal. Left: Original data. Right: Bootstrapped data, for which all error trials were reassigned into correct trials (see Methods). Bimodality was confirmed in 11/12 animals for the original data, and in 9/12 animals for the bootstrapped data (for details, see the 'Secondary Performance Metrics' section of Methods), with two animals falling just below the criterion value for the bootstrapped data sets. This suggests that rhythmic fluctuations in task performance went beyond the presence of individual error trials.

b) Correlations of local PR scores with all other behavioural metrics. Upper panel: Same as Fig. 5b, summarizing correlation coefficients between local PR scores and the other six primary metrics across all 12 animals. Data were smoothed using a 25-trial sliding averaging window before computing correlations. Lower panel: Same for bootstrapped data sets (see A). Cross-measure correlations were largely preserved in the bootstrapped data sets, indicating that the relation between PR scores and the other behavioural metrics was also not dependent on individual error trials.

c) Heat plot of pairwise explained variance (R^2, see colour bar) between seven primary performance metrics. Correlations were computed per animal, and then pooled using the Fisher-Z transform. Grey boxes and red arrows highlight the cross-measure correlations of the PR score. Left panels: Correlations between raw single-trial measures. Right panels: Correlations for data smoothed using a 25-trial sliding averaging window. Upper row: Original data. Lower row: Bootstrapped data. Cross-measure correlations derived

from the raw data (left panels) were confined entirely to those that would be predicted from mathematical necessity: Hit index and target distance correlate because the hit index essentially classifies trials according to target distance. The fact that the shared variance between all other measures was close to zero confirmed that there is only negligible intrinsic mathematical dependence, particularly between the PR score and other performance measures. In contrast, when measurements were smoothed using a 25-trial sliding window (right panels), PR scores correlated with a variety of measures. The correlations largely remained for bootstrapped data (lower row), indicating that these patterns could not be attributed to individual error trials. The reason cross-measure correlations emerged when data were averaged over time was presumably because smoothed data better reflected the slow transitions between high and low alertness. This allowed us to ascertain that the correlated fluctuations of various performance measures (e.g. lick location and reaction time) across High- and Low-Alert states could not be explained by 'hidden' mathematical cross-measure dependencies, but reflected genuine coordinated changes in cognitive processing.

Figure S3

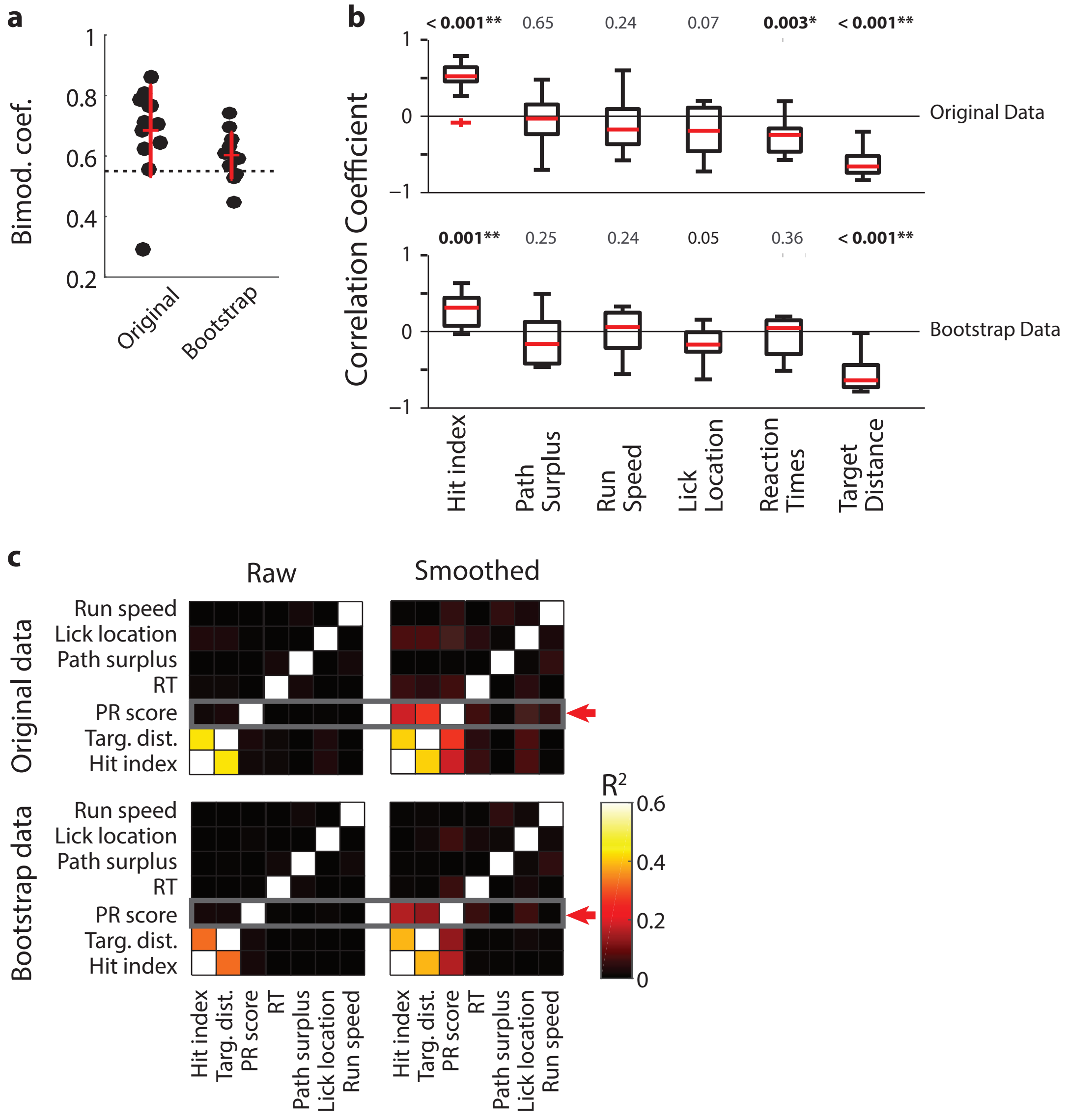

a
Bimod. coef.
1
0.8
0.6
0.4
0.2
Original
Bootstrap
b
Correlation Coefficient
< 0.001**
0.65
0.24
0.07
0.003*
< 0.001**
Original Data
0.001**
0.25
0.24
0.05
0.36
< 0.001**
Bootstrap Data
1
0
−1
Hit index
Path Surplus
Run Speed
Lick Location
Reaction Times
Target Distance
c
Raw
Smoothed
Original data
Bootstrap data
Run speed
Lick location
Path surplus
RT
PR score
Targ. dist.
Hit index
R^2
0.6
0.4
0.2
0

Figure S4: Distributions of High- and Low-Alert states in four example sessions

a) Progression of all trials beginning at training stage 5, classified as High- and Low-Alert, for four animals (for Mouse ID, see inset on right). For some animals (e.g. upper two panels) High-Alert and Low-Alert states are distributed quite evenly, for some (e.g. third panel) High-Alert states seem to occur increasingly as training progresses, and for others (e.g. bottom panel), High-Alert states always seem to be shorter than Low-Alert states.

b) Distribution of durations of High-Alert (black) and Low-Alert (grey) states for four mice, resulting from the progression of High-Alert and Low-Alert states shown in A. Left column: Durations expressed in trials. Right column: Same durations expressed in minutes (see Fig. 5c for the same distribution of durations, but pooled across all animals).

Figure S4

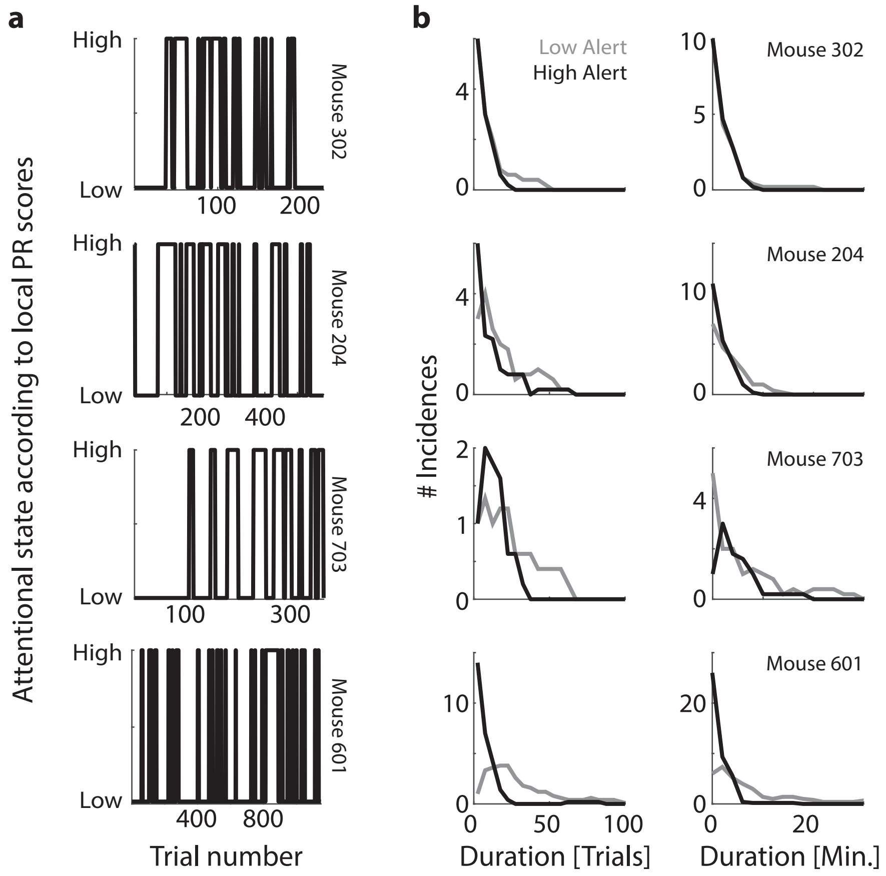
a
High
Low
Mouse 302
100
200
Mouse 204
200
400
Mouse 703
100
300
Mouse 601
400
800
Trial number
Attentional state according to local PR scores
b
Low Alert
High Alert
Incidences
Duration [Trials]
Duration [Min.]
0
50
100
20
10
5
4
2
1

Figure S5: High-Alert states and Cued-Attention states improve performance independently but to similar extents - particularly for difficult stimuli

a) Performance change from non-cued/low-alert to cued/high-alert trials, based on all sessions from training stage 6. Black: Performance change for High-Alert trials (n = 57 sessions in 12 animals). Blue: Performance change for accuracy incentive (n = 30 sessions from 6 animals). Red: Performance change for speed incentive (n = 27 sessions from 5 animals). Rectangle: 1st and 3rd quartile. Centre line: Median. Whiskers: 1st and 9th decile. Crosses: Outliers. Asterisks: Statistical significance of performance changes based on a Repeated-Measures ANOVA (*family-wise $p < 0.05$; ** family-wise $p < 0.01$ after Dunn-Sidak correction for multiple comparisons across six metrics). Three upper panels show the three primary performance metrics most improved by accuracy cuing. Lower panels show the three primary metrics most improved by speed cuing. As expected, hit index, target distance and path surplus improved particularly for accuracy-cued animals, while speed-cued animals produced faster reaction times and running speeds. Interestingly, they even licked earlier, possibly in order to access the reward as quickly as possible. Spontaneous High-Alert states acted more broadly (see Fig. 5), improving hit index and target distance, but also producing earlier licking and faster running.

b) Left panel: Change in CL index from non-cued to cued trials, split across all stimulus difficulties from ΔOri = 5° to ΔOri = 90°. Lines: Mean across animals. Error bars: SEM. Blue: Accuracy incentive (n = 6 animals). Red: Speed incentive (n = 5 animals). Stars indicate statistical significance of the change in CL index (*$p < 0.05$; **$p < 0.01$; for details of statistical testing, see Supp. Methods). Right panel: Same for High-Alert trials (black). As already indicated by the summary plot in Figure 6d, the CL index highlights similar increases of performance capacity for all three conditions (accuracy cue, speed

cue, and high alertness), irrespective of performance style. Performance improves particularly in difficult trials, likely due to the fact that for easy stimulus difficulties, animals already operate close to optimum irrespective of attentional state.

c) Scatter plot of average changes in CL index per animal, for spontaneous high-alert states (x-axis) versus cued trials (y-axis). Blue: Animals receiving the accuracy incentive (n = 6). Red: Animals receiving the speed incentive (n = 5). Performance improved for both high-alert and cued-attention states, as evidenced by the fact that data points are clustered in the lower left. However, some animals were more strongly improved by cuing (data points in lower right triangle), while others improved more strongly during high-alert states (data points in upper left triangle).

Figure S5

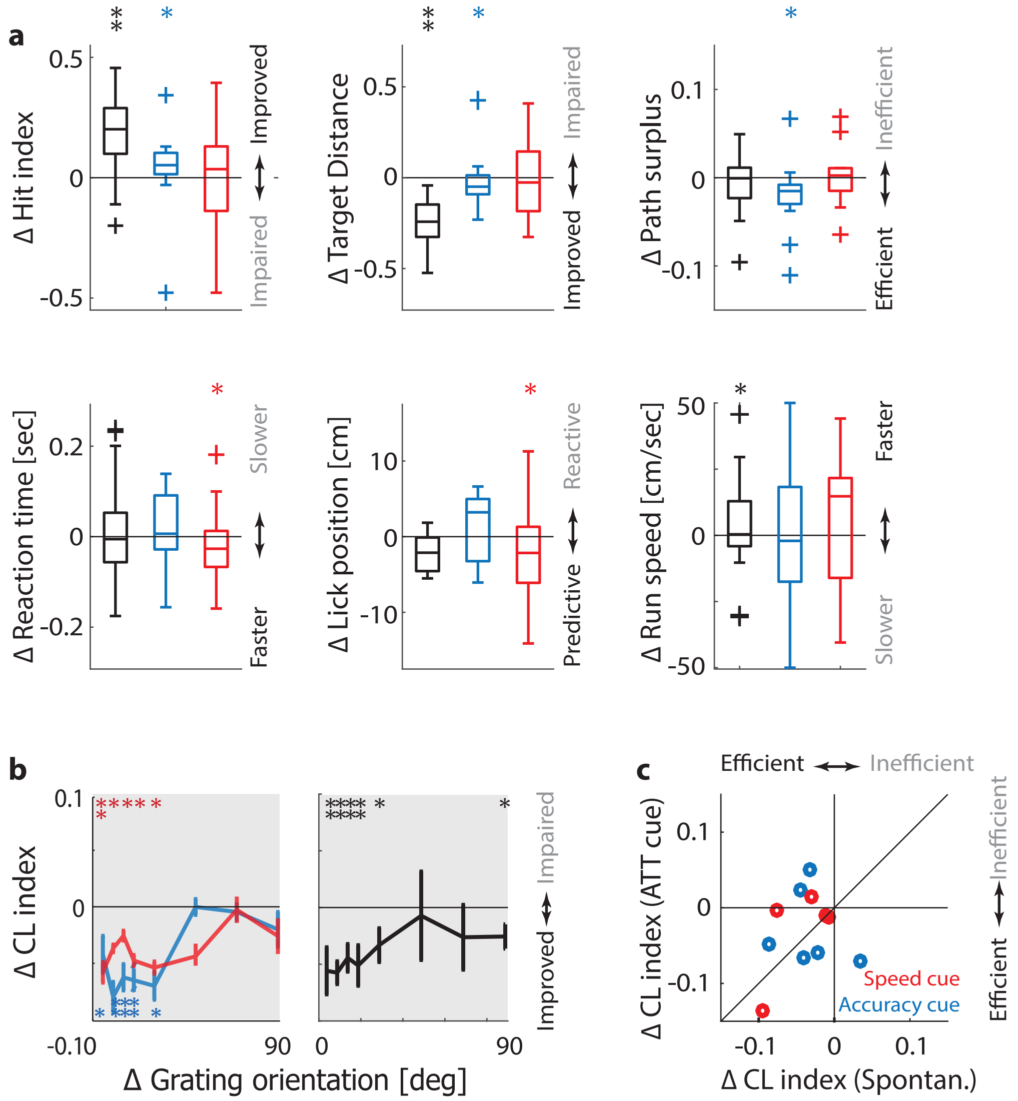

a
Δ Hit index
Improved
Impaired
Δ Target Distance
Impaired
Improved
Δ Path surplus
Inefficient
Efficient
Δ Reaction time [sec]
Slower
Faster
Δ Lick position [cm]
Reactive
Predictive
Δ Run speed [cm/sec]
Faster
Slower
b
Δ CL index
Impaired
Improved
Δ Grating orientation [deg]
c
Efficient
Inefficient
Δ CL index (ATT cue)
Speed cue
Accuracy cue
Δ CL index (Spontan.)

Figure S6: Speed-accuracy trade-off is reduced by task expertise, high alertness and cued attention

a) Discrimination threshold as a function of average reaction time, computed over the same trials. Dots: Animals. Dashed lines: Average discrimination threshold and reaction time across animals. In theory, high discrimination thresholds (see Fig. 4c) could result partially from prioritizing response speed rather than from low visual acuity. However, the correlation between discrimination thresholds and reaction times was marginally positive ($r = 0.28$; $p = 0.44$ for $n = 11$ animals), indicating that faster responses were linked to more, rather than less, accurate orientation discrimination. This seemed to contradict a large body of work demonstrating speed-accuracy trade-offs in species from humans to insects [20-24].

b) Distribution of correlation coefficients between reaction times and four indices of response accuracy (hit index, target distance, PR score and path surplus). Rectangle: 1st and 3rd quartile. Centre line: Median. Whiskers: 1st and 9th decile. Crosses: Outliers. Correlations were computed per training session. All sessions from training stage 4 onwards were included ($n = 73$ sessions in 12 animals). Numbers above the box plots denote the probability with which the distribution deviates from zero (* group-wise $p < 0.05$; ** group-wise $p < 0.01$ after Dunn-Sidak correction for multiple comparisons). The relation between reaction times and path surplus is highlighted in red because it is the only one that indicates a speed-accuracy trade-off: Reaction times correlated positively with target distance (58/73 correlation coefficients > 0; t-test for difference from zero: $p < 0.001$; $t = 4.90$; $df = 72$; see Supp. Table S1; mean $r = 0.08$), and negatively with hit index (61/73 correlation coefficients < 0; $p = 0.002$; $t = -5.02$; $df = 72$; mean $r = -0.09$) and PR score (54/73 correlation coefficients < 0; $p = 0.008$; $t = -2.67$; $df = 72$; mean $r = -0.09$). In other words, animals that responded faster also achieved better accuracy. In contrast, reaction times correlated negatively with path surplus (54/73 correlation

coefficients ≤ 0; $p = 0.013$; $t = -2.85$; $df = 72$; mean $r = -0.12$) - earlier reaction times were followed by longer running trajectories. The reason for this difference is presumably that the initial response, which generates the reaction time, and the moment of reaching the target (or distractor) occur several seconds apart, leaving time for corrections. Thus, the first stimulus response and the final target choice are not directly competing for cognitive resources. Since hit index, target distance and PR score reflect the final target choice, they require no trade-off. Path surplus on the other hand is based on the entire running trajectory starting at the initial response. Thus, at the initial choice of running direction, there is a trade-off: When animals respond quickly, running direction needs to be corrected more frequently, leading to suboptimal paths.

c) Average path surplus per animal, pooled across all sessions beginning at training stage 5, as a function of the average reaction time for the same sessions. Dots: 12 animals. Dashed lines: Average reaction time and path surplus across all animals. Based on the 'performance quadrant' they fall in, animals can be classified as high, low, 'impulsive' (fast but inaccurate), or 'thorough' (precise but slow) performers. Faced with the trade-off between reaction times and path surplus ($r = -0.22$; $p = 0.48$; $n = 11$ animals; see also Fig. S6b), different animals seemed to prioritise speed and path accuracy differently: While some were genuine low performers in both, others (labelled 'impulsive performers') seemed to require path correction because they responded quickly. Similarly, there was a split between genuine high performers and 'thorough performers' who produced accurate paths at the expense of speed. This classification can be a helpful tool, e.g. to identify animals according to visual acuity irrespective of performance strategy, or to find neural correlates of different behavioural styles (e.g. thorough versus impulsive).

d) Training reduces speed-accuracy trade-off. Left panel: Dots: Average reaction time and path surplus per session for three animals (see inset ID numbers). Warmer dot colours denote sessions later in training (starting from stage 5). Grey lines connect consecutive sessions. Dashed lines: Average reaction time and path surplus across all sessions and animals. Right panel: Same for another set of three animals. All animals show a general improvement in the speed-accuracy trade-off over time, with performance moving towards the 'high performance' quadrant (lower left). While some animals (e.g. 302, 703) improve in both speed and path accuracy, others improve mainly their response speed (e.g. 204, 401), or improve first path accuracy and then speed (501).

e) Alertness and cued attention reduce speed-accuracy trade-off per animal. Left panel: Relation between average reaction time and path surplus per animal, for Low-Alert and High-Alert trials. Arrow origin: Average reaction time and path surplus for Low-PR trials. Arrow point: Same for High-PR trials. Blue: Animals that mainly improve in terms of path surplus. Red: Animals that mainly improve in terms of speed. Grey: Unimproved animals. Dashed lines: Average reaction time and path surplus across all sessions and animals, starting from training stage 5. Note that animals with close-to-optimal path accuracy improve their response speed more consistently than other animals. Right panel: Same for cued attention. Arrow origin: Averages per animal for on-cued trials. Arrow point: Cued trials. Blue: Animals trained with the Accuracy incentive. Red: Animals trained with the Speed incentive. While cue-dependent performance changes seem smaller than those related to spontaneous fluctuations of alertness, the performance priority (speed or accuracy) generally matches the cuing incentive.

f) Alertness and cued attention reduce speed-accuracy trade-off per session. Left panel: Distributions of correlation coefficients between reaction times and path surplus,

computed individually for all sessions from training stage 5. Black: Correlations computed only for the High-Alert trials in a session. Grey: Correlations computed for Low-Alert trials. Stars denote the probability of the distribution being statistically different from zero (* $p < 0.05$; ** $p < 0.01$; Kolmogorov-Smirnov-Test; see Methods). Right panel: Same for cued attention (pooled across Accuracy and Speed cues). As predicted by the trade-off between speed and path accuracy explored in panels B-E of this figure, the distributions are generally skewed towards negative correlation coefficients between reaction time and path surplus. However, the correlations based on low-alert and non-cued trials differ significantly from zero, while correlations for high-alert and cued trials do not. This suggests that during high-alert and cued trials, there is less of a trade-off between response speed and accuracy, with responses being both fast and accurate (with the effect being somewhat stronger for spontaneous than cued changes in attention). This difference indicates that during high alertness or cued attention, cognitive capacity is increased, making behavioural trade-offs unnecessary.

Figure S6

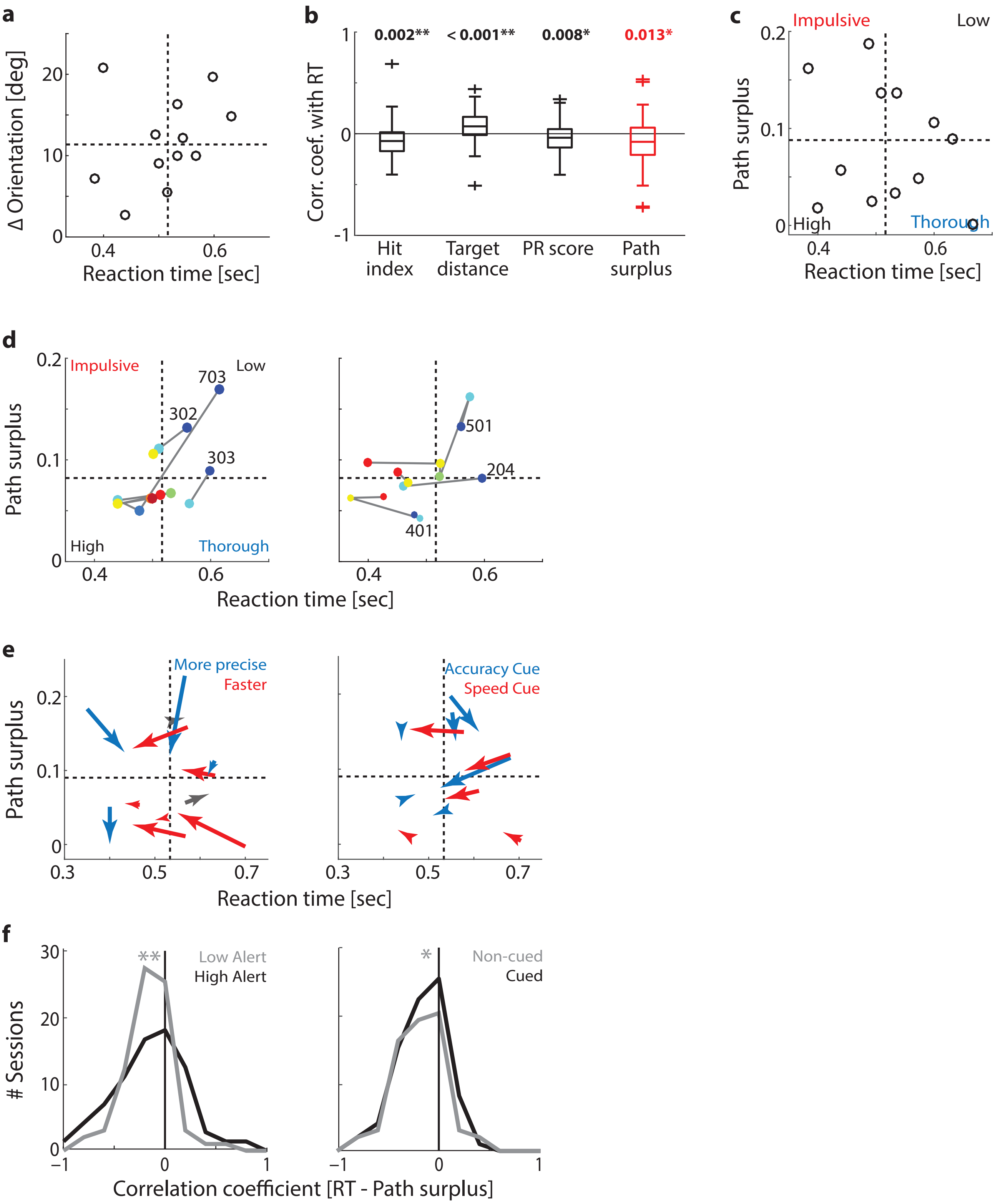
a
Δ Orientation [deg]
Reaction time [sec]
b
Corr. coef. with RT
0.002**
< 0.001**
0.008*
0.013*
Hit index
Target distance
PR score
Path surplus
c
Impulsive
Low
High
Thorough
Path surplus
Reaction time [sec]
d
Impulsive
Low
High
Thorough
703
302
303
501
204
401
Path surplus
Reaction time [sec]
e
More precise
Faster
Accuracy Cue
Speed Cue
Path surplus
Reaction time [sec]
f
Low Alert
High Alert
Non-cued
Cued
Sessions
Correlation coefficient [RT - Path surplus]

Figure S7: sATT and cATT score can be dissociated from visual acuity and learning ability

a) sATT score is independent of visual acuity and learning speed. Left panel: sATT score as a function of discrimination threshold (n = 12 animals). r: Correlation coefficient. Right panel: Same for sATT score as a function of trials required to reach training stage 5. For both correlations, $p > 0.2$.

b) Same as A for cATT score instead of sATT score. For both correlations, $p > 0.2$.

Figure S7

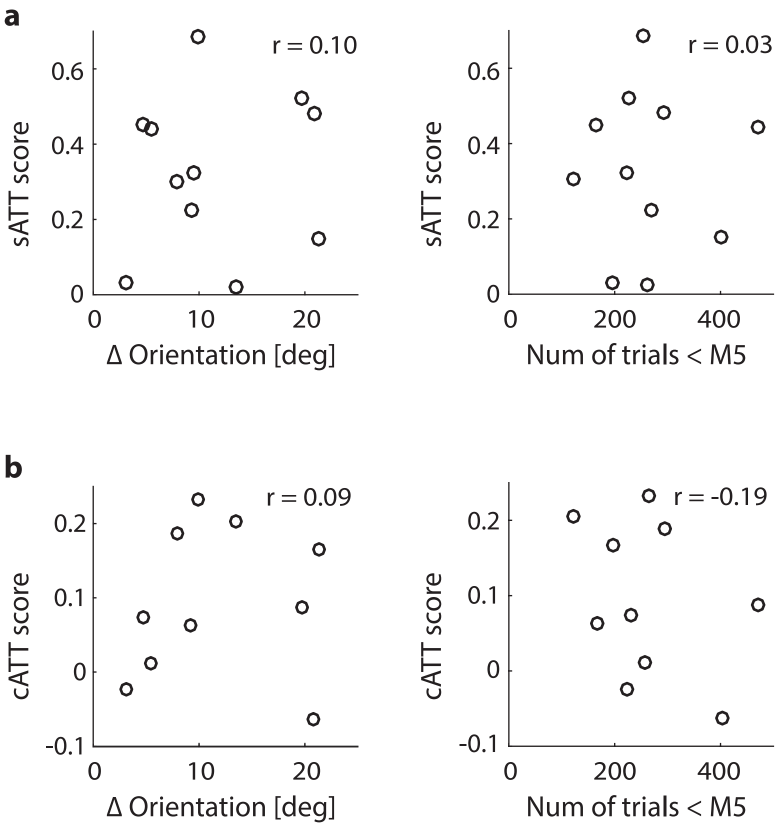
a
r = 0.10
sATT score
Δ Orientation [deg]
r = 0.03
sATT score
Num of trials < M5
b
r = 0.09
cATT score
Δ Orientation [deg]
r = -0.19
cATT score
Num of trials < M5

Supplementary Table S1 – Summary of statistical tests

For details on choice and conduct of statistical tests, please see last section of Methods. For Kolmogorov-Smirnov tests, we show the KS statistic, i.e. the maximum absolute difference between cumulative distributions, as a measure of effect size. Section G of the table shows a two-sided confidence interval (for α = 0.05) based on the z distribution that the measured performance changes per ΔOri were evaluated against (see Methods).

<table>
<tr><th>Figure</th><th>Data structure / Distribution</th><th>Statistical test</th><th>Power /
Confidence intervals (CI)</th></tr>
<tr><td>5e</td><td>Normal</td><td>t-test for difference of distribution from zero;
Family-wise α = 0.05; Corrected α = 0.013;
One-tailed</td><td>CI (95%):
r(Hit index); r = -0.14 to -0.06
r(Targ.Dist.): r = 0.04 to 0.12
r(PR score): r = -0.08 to -0.01
r(Path Surp.): r = -0.17 to -0.04</td></tr>
<tr><td>6b</td><td>Normal</td><td>T-test for difference from zero;
Family-wise α = 0.05; Corrected α = 0.0102;
One-tailed</td><td>95% Confidence intervals:
<table>
<tr><th></th><th>CI Lower</th><th>CI Upper</th></tr>
<tr><td>r (Hit ind.)</td><td>r = 0.35</td><td>r = 0.64</td></tr>
<tr><td>r(Path sur.)</td><td>r = -0.18</td><td>r = 0.23</td></tr>
<tr><td>r(Speed)</td><td>r = =-0.27</td><td>r = 0.13</td></tr>
<tr><td>r(Lick loc.)</td><td>r = -0.38</td><td>r = 0.02</td></tr>
<tr><td>r(RT)</td><td>r = -0.40</td><td>r = -0.11</td></tr>
<tr><td>r(CL index)</td><td>r = -0.47</td><td>r = -0.20</td></tr>
<tr><td>r(Targ.Dist.)</td><td>r = -0.72</td><td>r = -0.48</td></tr>
</table></td></tr>
<tr><td>6f</td><td>Normal</td><td>t-test for dependent samples, α = 0.05;
One-tailed</td><td>CI (Δ vis. threshold LowPR - HighPR):
-0.9° to 14.5°</td></tr>
<tr><td>6g</td><td>Normal</td><td>t-test for dependent samples, α = 0.05;
One-tailed</td><td>CI (Δ RT LowPR - HighPR):
-40ms to 63ms</td></tr>
<tr><td>7b</td><td>Normal</td><td>t-test for dependent samples, α =</td><td>CI (Δ vis. threshold ATTminus - ATTplus):
-9.8° to 6.0° (Speed cue)
-1.8° to 8.3° (Accuracy cue)</td></tr>
</table>

<table>
<tr><td></td><td></td><td>0.05;
One-tailed</td><td></td></tr>
<tr><td>7c</td><td>Normal</td><td>t-test for dependent samples, α = 0.05;
One-tailed</td><td>CI (Δ RT ATTminus - ATTplus):
-13ms to 105ms (Speed cue)
-69ms to 82ms (Accuracy cue)</td></tr>
<tr><td>S4b</td><td>Normal</td><td>t-test for difference of distribution from zero; Family-wise α = 0.05;
Corrected α = 0.0102;
One-tailed</td><td>95% Confidence intervals (lower panel):
<table>
<tr><th></th><th>CI Lower</th><th>CI Upper</th></tr>
<tr><td>r (Hit ind.)</td><td>r = 0.13</td><td>r = 0.37</td></tr>
<tr><td>r(Path sur.)</td><td>r = -0.24</td><td>r = 0.08</td></tr>
<tr><td>r(Speed)</td><td>r = =-0.14</td><td>r = 0.18</td></tr>
<tr><td>r(Lick loc.)</td><td>r = -0.29</td><td>r = - 0.03</td></tr>
<tr><td>r(RT)</td><td>r = -0.32</td><td>r = 0.04</td></tr>
<tr><td>r(CL index)</td><td>r = -0.32</td><td>r = -0.07</td></tr>
<tr><td>r(Targ.Dist.)</td><td>r = -0.67</td><td>r = -0.46</td></tr>
</table></td></tr>
<tr><td>S6a</td><td>Sample size too small to determine distribution (4/5 animals for speed and accuracy cue, respectively)</td><td>Z-test for difference from zero;
α = 0.05;
One-tailed</td><td>CI of test (z) distribution:
<table>
<tr><th></th><th>Acc. cue</th><th>Speed cue</th></tr>
<tr><td>Hit index</td><td>± 0.21</td><td>± 0.17</td></tr>
<tr><td>Targ.Dist</td><td>± 0.17</td><td>± 0.13</td></tr>
<tr><td>Path surp.</td><td>± 0.04</td><td>± 0.04</td></tr>
<tr><td>RT</td><td>± 0.09</td><td>± 0.07</td></tr>
<tr><td>Run speed</td><td>± 14.74</td><td>± 12.30</td></tr>
<tr><td>Lick loc.</td><td>± 4.63</td><td>± 4.22</td></tr>
</table></td></tr>
<tr><td>S6b</td><td>Sample size too small to determine distribution (11 animals)</td><td>Z-test for difference from zero;
α = 0.05;
One-tailed</td><td>CI of test (z) distribution:
<table>
<tr><td>Hit index</td><td>± 0.16</td></tr>
<tr><td>Targ.Dist</td><td>± 0.11</td></tr>
<tr><td>Path surp.</td><td>± 0.07</td></tr>
<tr><td>RT</td><td>± 0.12</td></tr>
<tr><td>Run speed</td><td>± 19.17</td></tr>
<tr><td>Lick loc.</td><td>± 3.90</td></tr>
<tr><td></td><td></td></tr>
</table></td></tr>
<tr><td>S6c</td><td>Sample size too small to determine distribution (4/5 animals for speed and accuracy cue, respectively)</td><td>Z-test for difference from zero;
α = 0.05;
One-tailed</td><td>CI of test (z) distribution:
<table>
<tr><th>PR high</th><th>Acc. cue</th><th>Speed cue</th></tr>
<tr><td>± 0.11</td><td>± 0.06</td><td>± 0.04</td></tr>
</table></td></tr>
</table>

<table>
<tr>
<td>S7b</td>
<td>Normal</td>
<td>t-test for difference of distribution from zero;
Family-wise α = 0.05; Corrected α = 0.013;
One-tailed</td>
<td>95% CIs of distribution of r(RT-Path surp.)
<table>
<tr><th></th><th>CI Lower</th><th>CI Upper</th></tr>
<tr><td>PR-high</td><td>r = -016</td><td>r = 0.05</td></tr>
<tr><td>PR-low</td><td>r = -0.12</td><td>r = -0.01</td></tr>
<tr><td>ATTplus</td><td>r = =-0.16</td><td>r = 0.03</td></tr>
<tr><td>ATTminus</td><td>r = -0.11</td><td>r =- 0.01</td></tr>
</table>
</td>
</tr>
</table>
